# Seminiferous tubule–inspired coaxial bioprinting–derived extracellular vesicles restore Leydig cell steroidogenesis through modulation of Wnt4/β-catenin signaling

**DOI:** 10.1186/s12951-026-04574-0

**Published:** 2026-05-21

**Authors:** Jialin Wu, Jianwei Chen, Yiran Tao, Qiang Guo, Jiayu Huang, Yi Zhang, Tianyou Zhang, Zijun Mo, Dejuan Wang, Tao Xu, Jianguang Qiu

**Affiliations:** 1 Department of Urology, The Sixth Affiliated Hospital, Sun Yat-sen University, No 26 Yuancun Erheng Road, Guangzhou, 510655 China; 2 Biomedical Innovation Center, The Sixth Affiliated Hospital, Sun Yat-sen University, Guangzhou, 510655 China; 3 Research and Development Department(R&D), Qingyuanzhixin（shenzhen） Biotechnology Co., LTD, 518000 Shenzhen, China; 4 Center for Bio-intelligent Manufacturing and Living Matter Bioprinting, Research Institute of Tsinghua University in Shenzhen, Tsinghua University, Shenzhen, 518057 China; 5 Department of Research and Development, Huaqing Zhimei (Shenzhen) Biotechnology Co., Ltd, Shenzhen, 518107 Guangdong Province China

## Abstract

Chemotherapy-associated gonadotoxicity compromises Leydig cell steroidogenesis and leads to testosterone deficiency, yet repair is hindered by the testicular barrier–immune niche and by limited manufacturability of biologics. Inspired by the tubular architecture of seminiferous tubules, we develop a coaxial bioprinting–enabled encapsulation culture that generates continuous core–shell hydrogel microfibers for high-density 3D culture of human umbilical cord MSCs (hUMSCs) and continuous conditioned-medium harvesting, producing seminiferous tubule–inspired coaxial bioprinting–derived extracellular vesicles (STi-EVs). We directly benchmark STi-EVs against conventional 2D culture–derived EVs (2D-EVs) in a 4-hydroperoxycyclophosphamide (4-HC)–injured TM3 model and a cyclophosphamide-induced mouse model. Across cellular fitness, re-engagement of steroidogenic programs, and testosterone output, STi-EVs consistently outperform 2D-EVs, while more effectively alleviating testicular histopathology and restoring serum testosterone in vivo. Transcriptomics prioritize Wnt signaling and nominate Wnt4; orthogonal validation and Wnt4 gain- and loss-of-function perturbations, and β-catenin stabilization experiments support that modulation of Wnt4/β-catenin signaling functionally contributes to STi-EV-mediated steroidogenic restoration. Collectively, this work couples a scalable extracellular vesicles (EV) manufacturing paradigm with a mechanism-informed repair strategy for endocrine sequelae after alkylating chemotherapy.

**Graphical Abstract:**

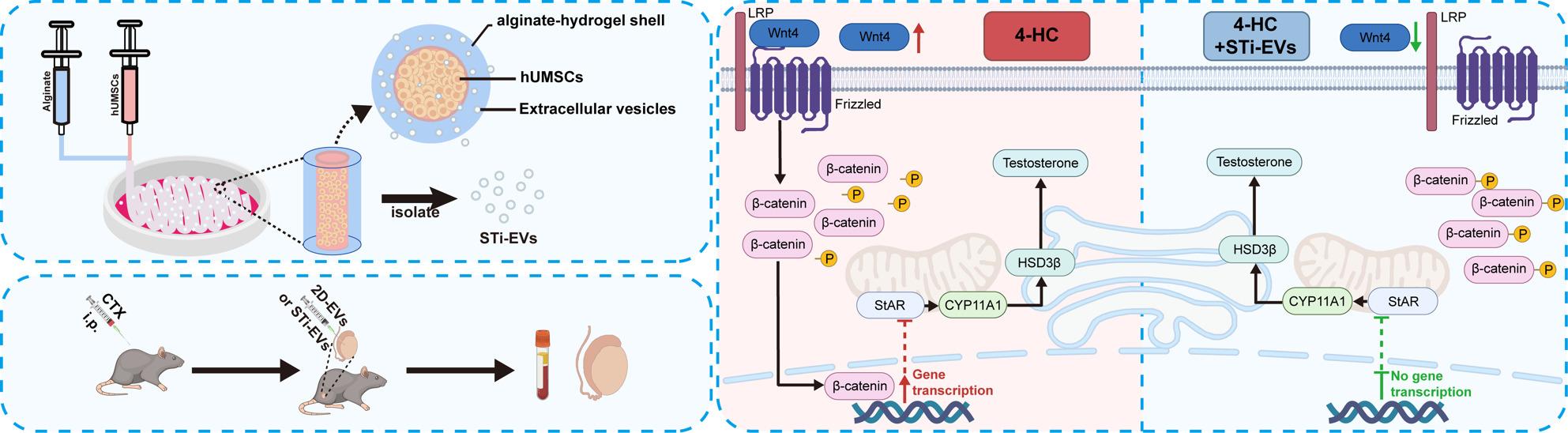

**Supplementary Information:**

The online version contains supplementary material available at 10.1186/s12951-026-04574-0.

## Introduction

Testicular disorders and therapy-related gonadotoxicity remain difficult to treat because the testis is protected by a highly specialized barrier–immune niche.[1–3] The blood–testis barrier (BTB), formed primarily by Sertoli cell junctional complexes, compartmentalizes the seminiferous epithelium and restricts access of circulating agents to the adluminal environment, thereby limiting drug delivery and complicating repair.[4] Beyond physical restriction, the immunologically privileged interstitial milieu is tightly regulated; its perturbation can amplify inflammatory damage and impair fertility.[2, 3] Importantly, the interstitium also harbors Leydig cells, the principal endocrine source of testosterone, such that gonadotoxic insults frequently extend beyond spermatogenic disruption to compromise steroidogenic capacity and systemic androgen homeostasis.[5, 6] Although stem cell–based approaches have been explored, their translation is constrained by limited in vivo persistence/engraftment, batch variability, and manufacturing and safety burdens.[7] In contrast, mesenchymal stem cell–derived extracellular vesicles (MSC-EVs) offer a cell-free modality that can recapitulate key paracrine functions via transfer of bioactive cargo, with a comparatively favorable safety and storage profile.[8] Consistently, EV-based interventions have shown benefit in diverse testis-injury contexts, including preservation of BTB function in aging models and mitigation of torsion–detorsion injury, supporting their promise for testicular homeostasis restoration.[9, 10].

A parallel barrier to clinical translation is manufacturability, especially for indications that may require repeated dosing. Architecturally, the testis is dominated by seminiferous tubules, suggesting that stable three-dimensional confinement in a tubule-like geometry may facilitate high-density culture while enabling controllable and continuous recovery of secreted products.[11–13] Inspired by the seminiferous tubule architecture at a strictly structural level, we established a coaxial bioprinting–based encapsulation culture to generate tubular core–shell hydrogel constructs that provide a cytocompatible 3D milieu and stable spatial confinement (with permissive mass transport/nutrient diffusion and mechanical protection), thereby sustaining high cell viability and secretory activity while enabling high-density 3D culture and continuous conditioned-medium harvesting. A related coaxial microfiber platform has been reported to markedly increase EV yield while reducing space, labor, time, and cost, thereby addressing a key bottleneck in scalable EV manufacturing.[14] Using human umbilical cord MSCs (hUMSCs), a scalable producer cell source with robust paracrine capacity, we generated EV in this system and termed them seminiferous tubule–inspired coaxial bioprinting–derived EVs (STi-EVs).[15, 16] Importantly, 3D culture can remodel EV cargo and functional phenotypes, linking scalable production with testable bioactivity rather than yield alone.

Cyclophosphamide (CTX), a prototypical alkylating agent, is extensively used as a broad-spectrum anticancer drug and immunosuppressant in malignancies, autoimmune disorders, and selected nephropathies. As survival improves, chemotherapy-associated gonadotoxicity has emerged as a clinically meaningful late effect, particularly among children, adolescents, and young adult cancer survivors, presenting as impaired spermatogenesis, testosterone insufficiency, and sexual dysfunction.[17, 18] Testosterone biosynthesis is indispensable for spermatogenesis, male fertility, and sexual function, yet chemotherapeutics can compromise testicular integrity, including by disrupting the blood–testis barrier, and alkylator toxicity often scales with cumulative dose and treatment duration.[17, 19] Because interstitial Leydig cells are the dominant source of testicular testosterone, Leydig cell dysfunction represents a clinically relevant but frequently underappreciated endocrine consequence of alkylator-based regimens. Although testosterone replacement therapy is commonly prescribed, it may suppress endogenous steroidogenesis and carries additional safety considerations, motivating safer, mechanism-guided strategies that restore intrinsic steroidogenic capacity.[20–22] These considerations motivate safer, mechanism-guided strategies that preserve or reconstitute Leydig cell steroidogenesis.

Mechanistically, the signaling circuitry connecting alkylating stress to Leydig cell steroidogenic failure remains incompletely understood.[23] Canonical Wnt/β-catenin signaling is a conserved regulator of tissue homeostasis with pivotal roles in gonadal biology; within this network, Wnt4 is a prominent ligand node that can engage canonical β-catenin–dependent transcriptional programs and has been implicated in maintaining testicular homeostasis and constraining steroidogenic gene expression when aberrantly activated.[24–26] These observations nominate Wnt4/β-catenin as a plausible mechanistic hub for chemotherapy-associated endocrine dysfunction. However, Wnt4/β-catenin has been studied predominantly in developmental and homeostatic pathway balance, and whether this axis can be therapeutically leveraged to reverse acquired Leydig testosterone deficiency after gonadotoxic stress remains largely unexplored.[27, 28] Therefore, we prioritized a Wnt4-centered hypothesis and tested it through transcriptome-guided candidate selection, multi-level validation, and Wnt4 gain- and loss-of-function perturbations.

**Fig. 1:**
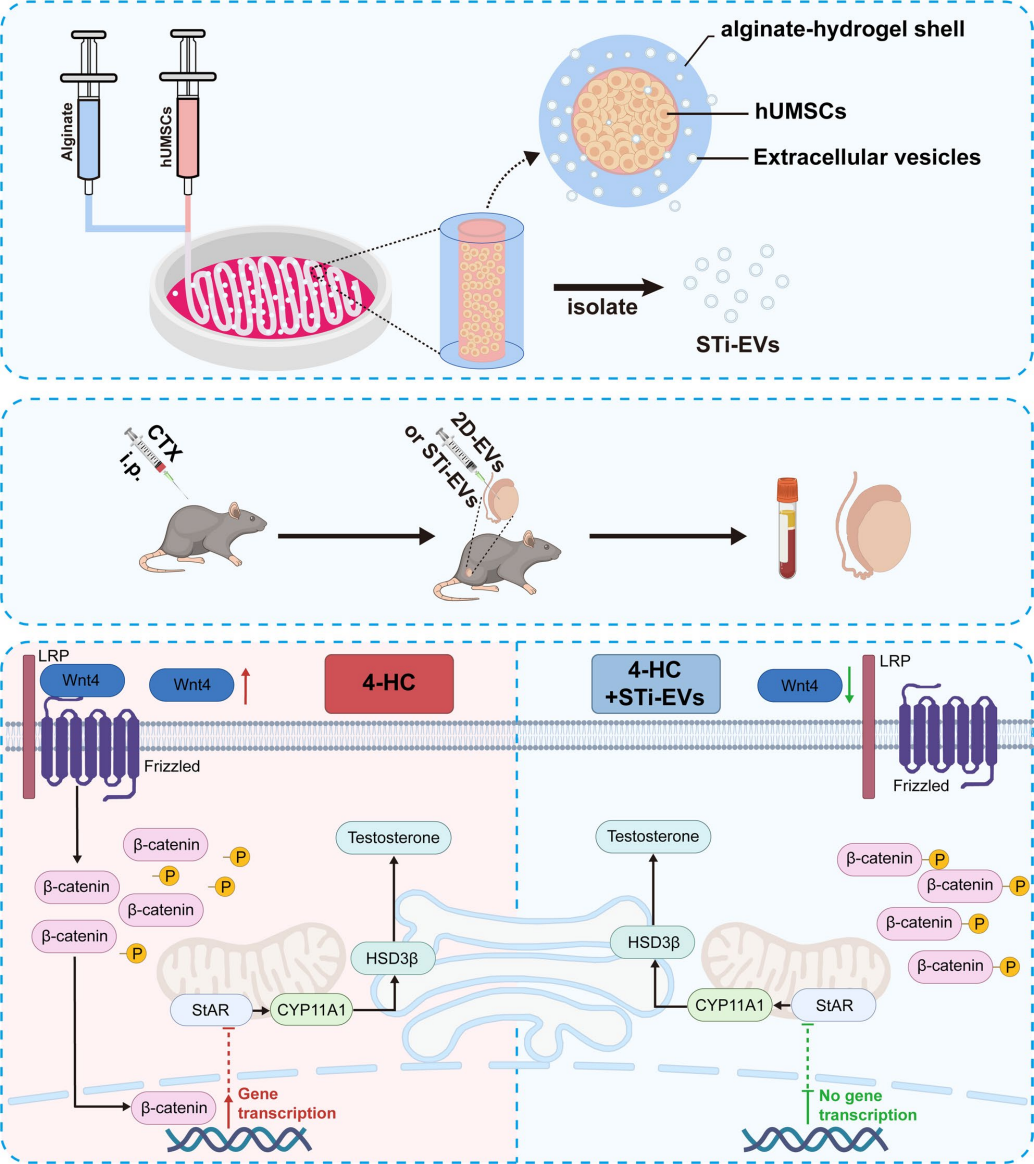
Schematic illustrates the production of STi-EVs. Therapeutic EV were delivered via intratesticular injection in a CTX-induced gonadotoxicity model and evaluated alongside a 4-HC–injured TM3 system to quantify restoration of steroidogenesis and testosterone production. Mechanistically, chemotherapy-associated injury is associated with aberrant activation of Wnt4/β-catenin signaling and suppression of key steroidogenic nodes, whereas STi-EVs attenuate this signaling pattern and re-engage the steroidogenic program to improve testosterone output

Despite prior EV studies in testicular injury and prior 3D EV manufacturing approaches, an integrated framework that couples manufacturable EV supply with dose-matched endocrine potency benchmarking and pathway-level mechanistic validation remains lacking. Here, using the active CTX metabolite 4-hydroperoxycyclophosphamide (4-HC) in TM3 cells and a CTX-induced mouse model of chemotherapy-associated gonadotoxicity, we systematically evaluate whether STi-EVs restore Leydig cell steroidogenesis and testosterone output more effectively than conventional 2D culture–derived EV (2D-EVs) under dose-matched conditions. Importantly, coaxial, tubule-like confinement is used here as an upstream manufacturing architecture, enabling continuous harvest while supporting testable endocrine bioactivity. To this end, by integrating transcriptomic prioritization with multi-level in vitro and in vivo validation and Wnt4 gain- and loss-of-function perturbations, we test the hypothesis that modulation of Wnt4/β-catenin signaling contributes to STi-EV-mediated repair of endocrine dysfunction (Fig. 1). Collectively, this work couples a manufacturable EV production paradigm with a functionally supported pathway axis, providing a translationally relevant framework for endocrine sequelae following alkylating chemotherapy.

## Methods

### Cell culture

Human umbilical cord mesenchymal stromal cells (hUMSCs) were purchased from the Chinese Academy of Science and cultured in MSCM (Mesenchymal stem cell culture medium) with 10% FBS (fetal bovine serum) and 1% penicillin/streptomycin at 37 °C with 5% CO2. Mouse Leydig cells (TM3) were purchased from the Chinese Academy of Science, and cultured in DMEM/F12 medium containing 2.5% FBS, 5% horse serum and 1% penicillin/streptomycin at 37 °C and 5% CO2. To establish a 4-HC treatment model for TM3 cells, TM3 cells were exposed to different concentrations of 4-HC for 24 h, followed by replacement with fresh culture medium and the addition of 2D-EVs or STi-EVs for treatment. Unless otherwise specified, the extracellular vesicle dose used in cell experiments was 1 × 10^9 particles/mL. [29] All the cell lines were authenticated by STR profiling and tested clean for mycoplasma contamination.

### Coaxial bioprinting of seminiferous-tubule–inspired tubular core–shell microfibers (STi-Microfibers)

To emulate the tubular hierarchy of seminiferous tubules, a double-layer coaxial extrusion strategy was used to generate continuous core–shell microfibers. A representative coaxial configuration employed an inner-channel diameter of approximately 0.38 mm and an outer-channel diameter of approximately 1.1 mm, with the nozzle tip positioned in a 3% CaCl₂ crosslinking bath to enable rapid ionic gelation of the shell. The Shell phase is an alginate solution (tubule-wall–like matrix layer) and the core phase is high-density MSC suspension (lumen-like cellular core). Core and shell flow rates were independently regulated using syringe pumps; typical reference ranges were 15–30 mL/h for the shell and 3–10 mL/h for the core to ensure stable, continuous fibers and tunable lumen diameter/wall thickness. For comparability between platforms, an aligned collection scheme was used. 2D culture: MSCs were grown to 60%–80% confluency, rinsed 2–3 times with PBS (approximately 5 min each) to reduce serum protein/vesicle carryover, and then cultured in culture-conditioned medium for 48 h prior to supernatant collection.[14].

### Isolation and characterization of 2D-EVs and STi-EVs

For comparability between platforms, an aligned collection scheme was used. 2D culture: MSCs were grown to 60%–80% confluency, rinsed 2–3 times with PBS (approximately 5 min each) to reduce serum protein/vesicle carryover, and then cultured in culture-conditioned medium for 48 h prior to supernatant collection. STi-Microfibers culture: following fabrication, the culture-conditioned medium was collected directly from Microfibers every 48 h. All conditioned media were stored at −80 °C, and EV were extracted as soon as the supernatant was collected. Conditioned media were sequentially centrifuged at 300 × g (10 min), 2,000 × g (30 min), and 10,000 × g (30 min), followed by 0.22 μm filtration. EV were pelleted by ultracentrifugation at 100,000 × g (70 min, 4 °C), washed in PBS, and ultracentrifuged again at 100,000 × g (70 min). Final pellets were resuspended in PBS for downstream assays. All centrifugations were performed at 4 °C using a tabletop ultracentrifuge (Optima MAX-XP, Beckman Coulter, USA). Protein concentration of extracellular vesicles (3*10^10 particles, 100ul) was examined by BCA assay (CWBIO, Beijing, China) and subjected to western blot analysis for CD81 (ABclonal, A5270), CD63 (Abcam, ab68418), Calnexin (ABclonal, A15631), TSG101 (Abcam, ab125011), and Goat anti-Rabbit IgG Peroxidase Conjugated (Abcam). We observed the size and structure of extracellular vesicles with a transmission electron microscope (TEM) (Hitachi, 120 kV, Japan), and nanoparticle tracking analysis (NTA; Particle Metrix, Germany).

### Fluorescent labeling and cellular uptake

EV were fluorescently labeled (DiI) and co-incubated with TM3 cells. After 24 h, internalization was visualized by high-content fluorescence microscope with nuclei counterstained by DAPI and F-actin labeled to delineate cell boundaries.

### Live/dead staining

STi-microfibers were washed three times with 0.9% NaCl, incubated in 0.9% NaCl containing 2 µM calcein-AM and 8 µM PI (Beyotime, China) at 37 °C for 30 min in the dark, washed, and imaged by high-content fluorescence microscope. After the indicated treatments, cells were washed three times with PBS, incubated in PBS containing 2 µM calcein-AM and 2 µM PI at 37 °C for 30 min in the dark, washed, and imaged by a high-content fluorescence microscope.

### CCK-8 viability assay

For TM3 cell viability assay, TM3 cells were seeded into 96-well plates at 5 × 10^3 cells/well. After the indicated treatments, 10 µL of CCK-8 reagent was added to each well of a 96-well plate and incubated for approximately 2 h at 37 °C protected from light. Absorbance was recorded at 450 nm using a microplate reader.

### EdU incorporation assay

Following treatments, EdU labeling solution was added for 2 h, followed by fixation, permeabilization, click chemistry reaction, and DAPI counterstaining. EdU-positive nuclei were quantified with ImageJ.

### Scratch wound-healing assay

TM3 cells were seeded in six-well plates to 80% confluence, serum-starved overnight, scratched with 200 µL tips, rinsed with PBS, and cultured in conditioned medium. Images were taken at 0, 12, and 24 h and quantified with ImageJ.

### qRT-PCR

 RNA from TM3 cells or testis tissues was collected utilizing a total RNA kit (Omega) following the manufacturer’s instructions. cDNA was synthesized from the RNA using Evo M-MLV RT Kit with gDNA Clean for qPCR (Accurate Biology, China). Then qRT-PCR assays were performed using SYBR green (Accurate Biology). Relative gene expression normalized to GAPDH was calculated using the 2 ^-ΔΔCt method. All primers were synthesized by Tsingke (Beijing Tsingke Biotech Co., Ltd.) and listed in Table S1.

### Western Blot

Total protein was isolated from TM3 cells or tissue samples using RIPA lysis buffer (Beyotime, China) supplemented with a protease inhibitor cocktail (Solarbio, Beijing, China). Protein concentration was measured with a BCA kit (CWBIO). Equal amounts of protein were resolved on 10% SDS–PAGE gels and electrotransferred onto PVDF membranes (Millipore). Membranes were then blocked with 5% non-fat milk at room temperature and incubated with the indicated primary antibodies against GAPDH (Servicebio, GB12002), StAR (Proteintech, 67130-1-lg), CYP11A1 (GeneTex, GTX56293), Wnt4 (GenTex, GTX637638), β-catenin (CST, 8480 S) and Non-phospho (Active) β-Catenin (CST, 8814 S) diluted in antibody diluent at 4 °C overnight. After washing, membranes were incubated with HRP-conjugated secondary antibodies (Abcam) for 1 h at room temperature. Finally, the protein bands were visualized utilizing enhanced chemiluminescence reagents (GBCBIO, China) and quantified by ImageJ software.

### Immunocytochemistry

Cells grown on coverslips (or tissue cryosections/paraffin sections after antigen retrieval) were fixed with 4% paraformaldehyde at room temperature, rinsed with PBS, and permeabilized with 0.1% Triton X-100. Samples were then blocked with 5% bovine serum albumin (BSA) for 1 h to reduce nonspecific binding. Primary antibodies against HSD3β (Santa Cruz, sc-515120), Wnt4 were diluted in antibody diluent and incubated with samples at 4 °C overnight. After washing with PBST, samples were incubated with fluorophore-conjugated secondary antibodies for 1 h at room temperature in the dark. F-actin was counterstained with phalloidin, and nuclei were counterstained with DAPI. Image were taken using a high-content fluorescence microscope.

### Enzyme-linked immunosorbent assay (ELISA)

The protein expression levels of testosterone in cell supernatant and mouse serum were detected using a mouse testosterone enzyme-linked immunosorbent assay kit (MEIMIAN, MM-0569M1) according to the manufacturer’s instructions.

### Animal model

The mouse experiments were carried out according to protocols approved by the Institutional Animal Care and Use Committee (ACUC) of Shenzhen Top Biotech. Co., Ltd. (approval number: TO-1PZ-GM251211). The mice were housed at Top Biotech. Co., Ltd. (Shenzhen, China) under specific pathogen-free (SPF) conditions at 23 ± 2 °C ambient temperature with 40% humidity and a 12 h light/dark cycle. The male C57BL/6J mice (6–8 weeks, 20.0 ± 5.0 g, *n* = 24) were randomly allocated into the sham group, CTX model group, CTX +2D-EVs group, CTX + STi-EVs group (*n* = 6 per group) using the random number table method. A CTX model was established according to a previous study. The animals were acclimatized to the experimental conditions for one week prior to the commencement of the experiment. In order to establish a chemotherapy-related gonadotoxicity mouse model, mice were given intraperitoneal CTX at 60 mg/kg body weight each day over a 7-day period.[30] 2D- and STi-EVs were applied three times through intratesticular injection each time with 1 × 10^10 particles per testis on days 7, 10, and 13 [31, 32]. On day 21, we weighed the mice, collected blood, and removed their testes for various analyses.

### Histology evaluation and immunofluorescence staining

The testicular index was calculated by dividing the weight of the organ by the weight of the body. Mouse testis were fixed in 4% paraformaldehyde and embedded in paraffin. Paraffin was cut into sections and mounted on glass slides, deparaffinized with xylene, dehydrated through a graded series of ethanol, and stained with hematoxylin-eosin. The assessment of testicular injury was performed using the Johnsen’s score (JS) system in a blinded manner, which ranges from 0 (indicating absence of seminiferous epithelial cells and tubular sclerosis) to 10 (representing complete spermatogenesis), to evaluate the degree of spermatogenic impairment. For immunofluorescence staining, slide-mounted tissues were blocked with 5% BSA for 1 h and then incubated overnight at 4 ◦C with primary antibodies, followed by staining with corresponding secondary antibodies at room temperature. Cell nuclei were stained with DAPI. Images were captured using a high-content fluorescence microscope. The acquired images were quantified by ImageJ software.

### Plasmid and siRNA transfection

 Wnt4 cDNA overexpression plasmid and small interfering RNA (si-Wnt4) were purchased from Genecopoeia (Guangzhou, China). Three Wnt4-targeting siRNAs were designed using the supplier’s algorithm with basic off-target filtering. The corresponding sequences are provided in Table S2. TM3 cells were seeded to 80% confluence in 6-well plates in triplicate. Lipofectamine 3000 (Life Technologies) reagent was used to transfect Wnt4 plasmid or si-Wnt4 oligos into TM3 cells. Cell protein or RNA samples were harvested at 48 h after transfection and processed for western blot or qRT-PCR analysis.

### Statistical analysis

Data are presented as means ± SD (standard deviation). Statistical significance between two groups was assessed using Student’s t-test in GraphPad Prism (version 10.0). For comparisons among multiple groups, one-way ANOVA followed by Dunnett’s post hoc test was used. A two-sided *P* < 0.05 was considered statistically significant.

## Results

### Seminiferous tubule–inspired coaxial bioprinting enables scalable production and baseline characterization of STi-EVs

**Fig. 2:**
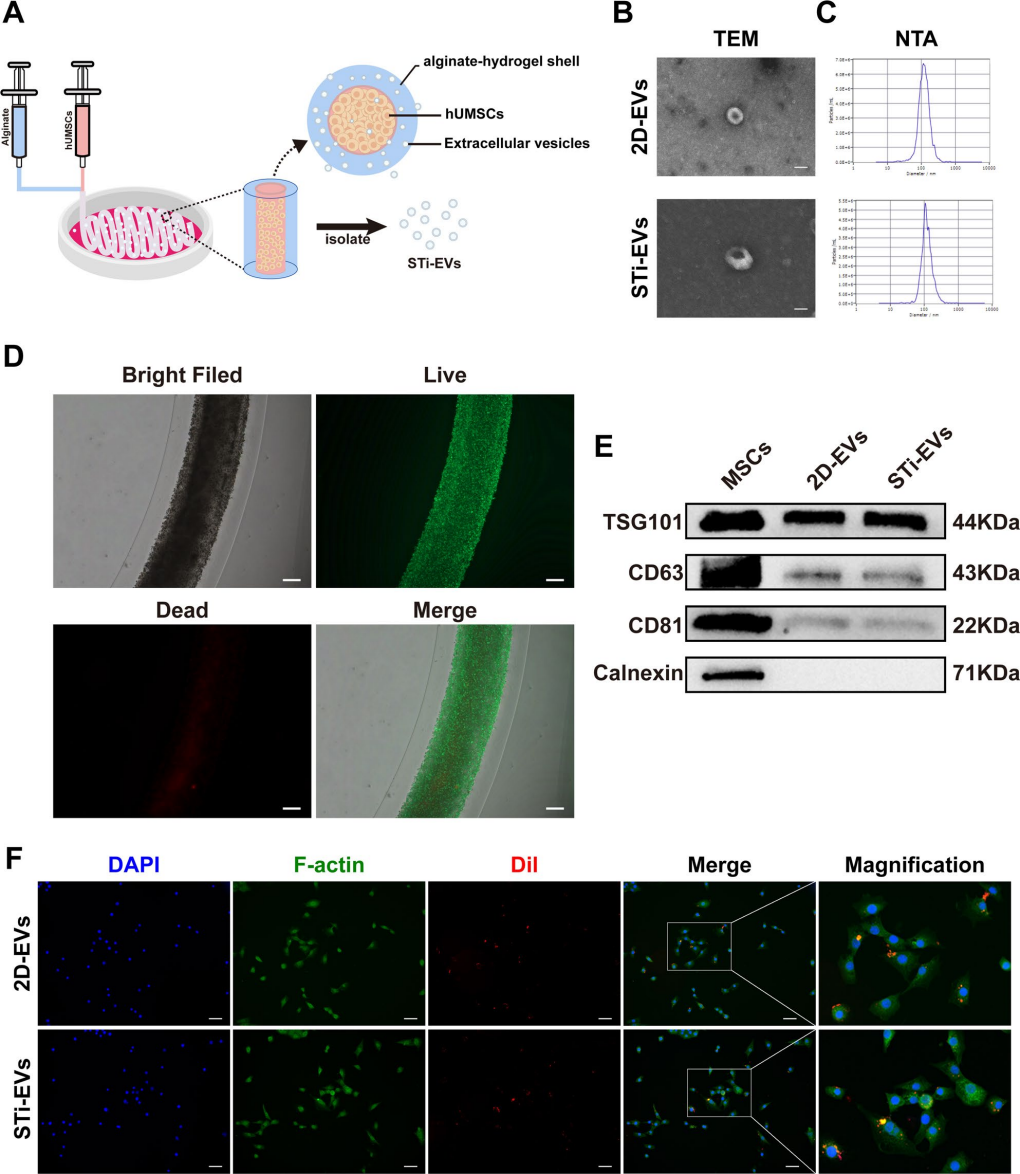
Seminiferous tubule–inspired coaxial bioprinting workflow and baseline characterization of STi-EVs. (**A**) Schematic of the seminiferous tubule–inspired coaxial bioprinting encapsulation culture used to generate tubular alginate core–shell constructs for high-density 3D culture and continuous conditioned-medium harvesting; EVs from conventional 2D monolayer culture were prepared in parallel as 2D-EVs. (**B**) Representative TEM images of 2D-EVs and STi-EVs. Scale bar = 50 nm. (**C**) Particle size distribution of 2D-EVs and STi-EVs measured by NTA. (**D**) Live/dead staining and bright-field images showing cell viability within the coaxially bioprinted core–shell constructs. Live cells, green; dead cells, red. Scale bar = 50 μm. (**E**) Western blot analysis of canonical EV markers in 2D-EVs and STi-EVs. (**F**) Representative images of TM3 cells after 24 h co-incubation with fluorescently labeled EV, showing cellular uptake. Nuclei were counterstained with DAPI (blue), F-actin was labeled in green, and EV are shown in red (DiI). Scale bar = 50 μm. *n* = 3 per group

To enable scalable EV production, we implemented a coaxial bioprinting–based core–shell hydrogel tubular construct whose cross-sectional geometry is seminiferous tubule–inspired in an architectural sense only, thereby supporting high-density 3D encapsulation culture of hUMSCs with continuous conditioned-medium harvesting to generate STi-EVs (Fig. 2A). EVs collected from conventional 2D monolayer culture were processed in parallel as 2D-EVs. Transmission electron microscopy showed the characteristic cup-shaped morphology for both 2D-EVs and STi-EVs, while nanoparticle tracking analysis revealed a predominant particle size distribution centered at approximately 100 nm (Fig. 2B–C). Live/dead staining together with bright-field imaging confirmed high cell viability within the coaxially printed constructs during culture (Fig. 2D). Western blot further validated EV identity by demonstrating canonical EV markers with minimal non-EV contamination (Fig. 2E). Finally, fluorescence tracing indicated efficient cellular uptake of both 2D-EVs and STi-EVs by TM3 cells after 24 h of co-incubation (Fig. 2F). Direct quantitative productivity analysis revealed that STi-EVs showed substantially higher productivity than 2D-EVs across all evaluated output metrics. Specifically, STi-EVs generated roughly three- to four-fold more particles than 2D-EVs when normalized by conditioned-medium volume, producer-cell number, and per-dish output over 48 h, indicating a consistently greater EV yield in the STi system (Fig. S8A–C). Having established that the coaxial platform supported viable high-density hUMSC culture and yielded EVs with canonical morphology, size distribution, marker expression, and recipient-cell uptake, we next asked whether this manufacturable EV source could functionally repair chemotherapy-associated Leydig cell injury. To address this question in a controlled and mechanistically tractable setting, we established a 4-HC–induced TM3 injury model as an in vitro surrogate of alkylating stress before comparing the restorative activities of dose-matched 2D-EVs and STi-EVs.

### 4-HC induces rapid steroidogenic dysfunction in TM3 cells

**Fig. 3:**
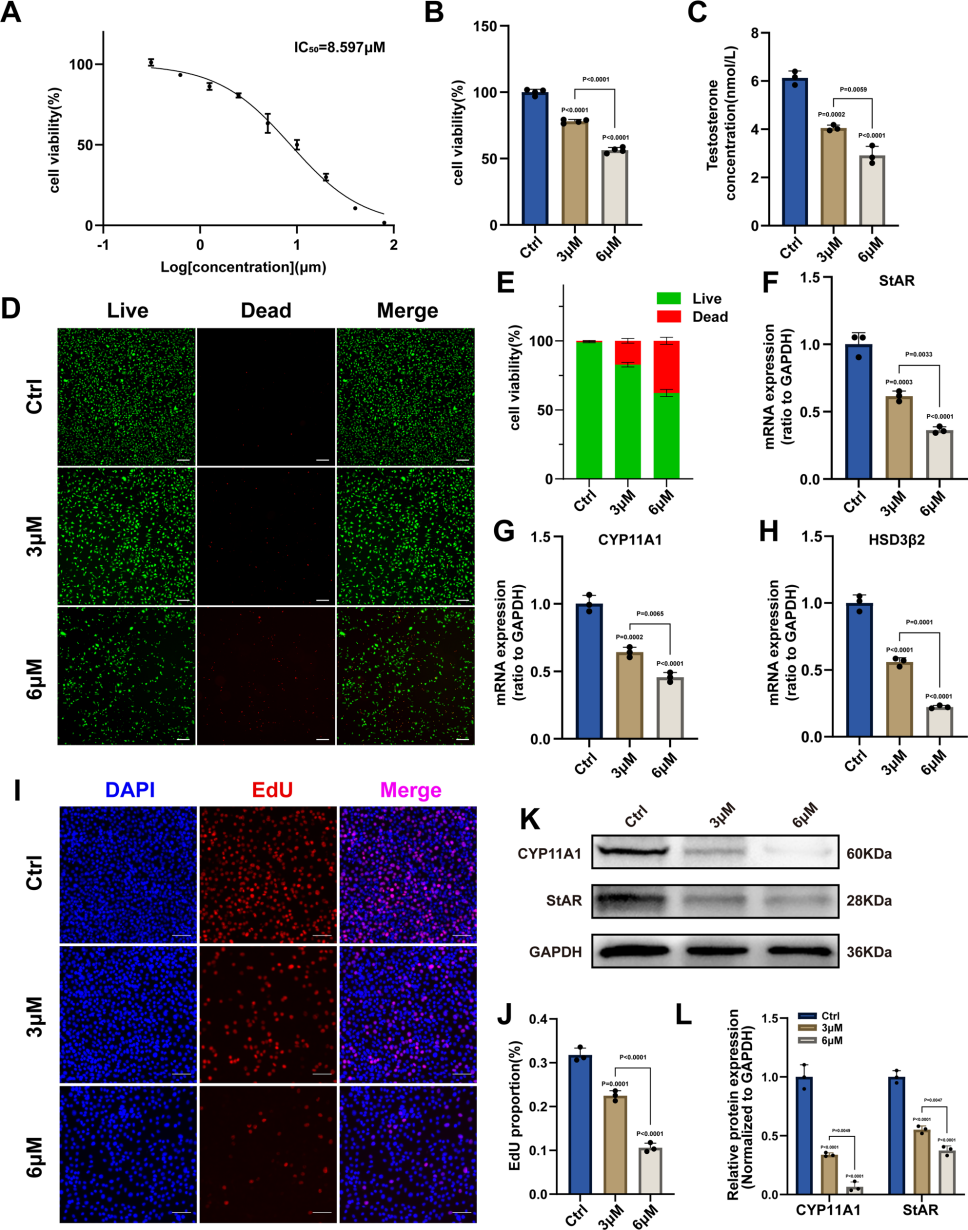
4-HC induces dose-dependent loss of viability and proliferation in TM3 cells and suppresses the steroidogenic program and testosterone output within 24 h. (**A**) Dose–response curve of TM3 cell viability after 24 h exposure to increasing concentrations of 4-HC, with the fitted IC₅₀ (8.597 µM). (**B**) TM3 cell viability at representative 4-HC concentrations (3 µM and 6 µM) after 24 h. (**C**) Testosterone concentration of TM3 cells after different treatments. (**D**) Representative Live/Dead staining of TM3 cells after 24 h treatment (live, green; dead, red; merged views shown). Scale bar = 100 μm. (**E**) Quantification of Live/Dead staining. (**F–H**) qPCR analysis of steroidogenesis-related genes (StAR, CYP11A1, and HSD3β2) across indicated 4-HC concentrations (normalized to GAPDH). (**I**) Representative EdU incorporation images showing proliferation after 24 h treatment (DAPI, blue; EdU, red; merged views shown). Scale bar = 50 μm. (**J**) Quantification of EdU-positive cells. (**K**) Western blot analysis of steroidogenic proteins (CYP11A1 and StAR; GAPDH as loading control). (**L**) Densitometric quantification of western blot. Statistical significance is indicated in the panels. Data are presented as mean ± SD. *n* = 3 per group

To simulate Leydig cell dysfunction induced by exposure to alkylating agent metabolites, we established a 4-HC treatment model in TM3 cells. TM3 cells were incubated with increasing concentrations of 4-hydroperoxycyclophosphamide (4-HC) for 24 h, followed by integrated readouts of viability, live/dead status, proliferation, and steroidogenic phenotypes. Dose–response modeling demonstrated a clear concentration-dependent reduction in viability, yielding a fitted IC₅₀ of 8.597 µM (Fig. 3A). At representative concentrations, 3 µM 4-HC reduced TM3 cell viability to 76.30 ± 2.99%, whereas 6 µM further decreased viability to 53.87 ± 4.86%, with a significantly greater reduction at 6 µM than at 3 µM (Fig. 3B). Live/Dead staining revealed a dose-dependent shift toward cell death (Fig. 3D), which was confirmed by quantitative analysis (Fig. 3E). In parallel, EdU incorporation assays showed a significant, dose-dependent suppression of proliferative capacity (Fig. 3I–J). We next interrogated the steroidogenic machinery. qPCR analysis demonstrated stepwise downregulation of core testosterone biosynthetic transcripts (StAR, CYP11A1, and HSD3β2) across increasing 4-HC concentrations (Fig. 3F–H). Consistently, western blot showed decreased CYP11A1 and StAR abundance with escalating 4-HC exposure, and densitometric quantification confirmed statistically significant differences (Fig. 3K–L). Functionally, testosterone output mirrored these molecular changes: supernatant testosterone progressively declined from 6.13 ± 0.28 nmol/L in controls to 4.05 ± 0.12 nmol/L at 3 µM and 2.92 ± 0.37 nmol/L at 6 µM (Fig. 3C), indicating rapid impairment of steroid production within 24 h. Because TM3 cell viability remained 76.30 ± 2.99% at 3 µM 4-HC, this concentration was selected for subsequent in vitro experiments to standardize the injury condition while preserving adequate cell viability for mechanistic interrogation.

### STi-EVs outperform 2D-EVs in restoring TM3 cell fitness and steroidogenesis after 4-HC injury

**Fig. 4:**
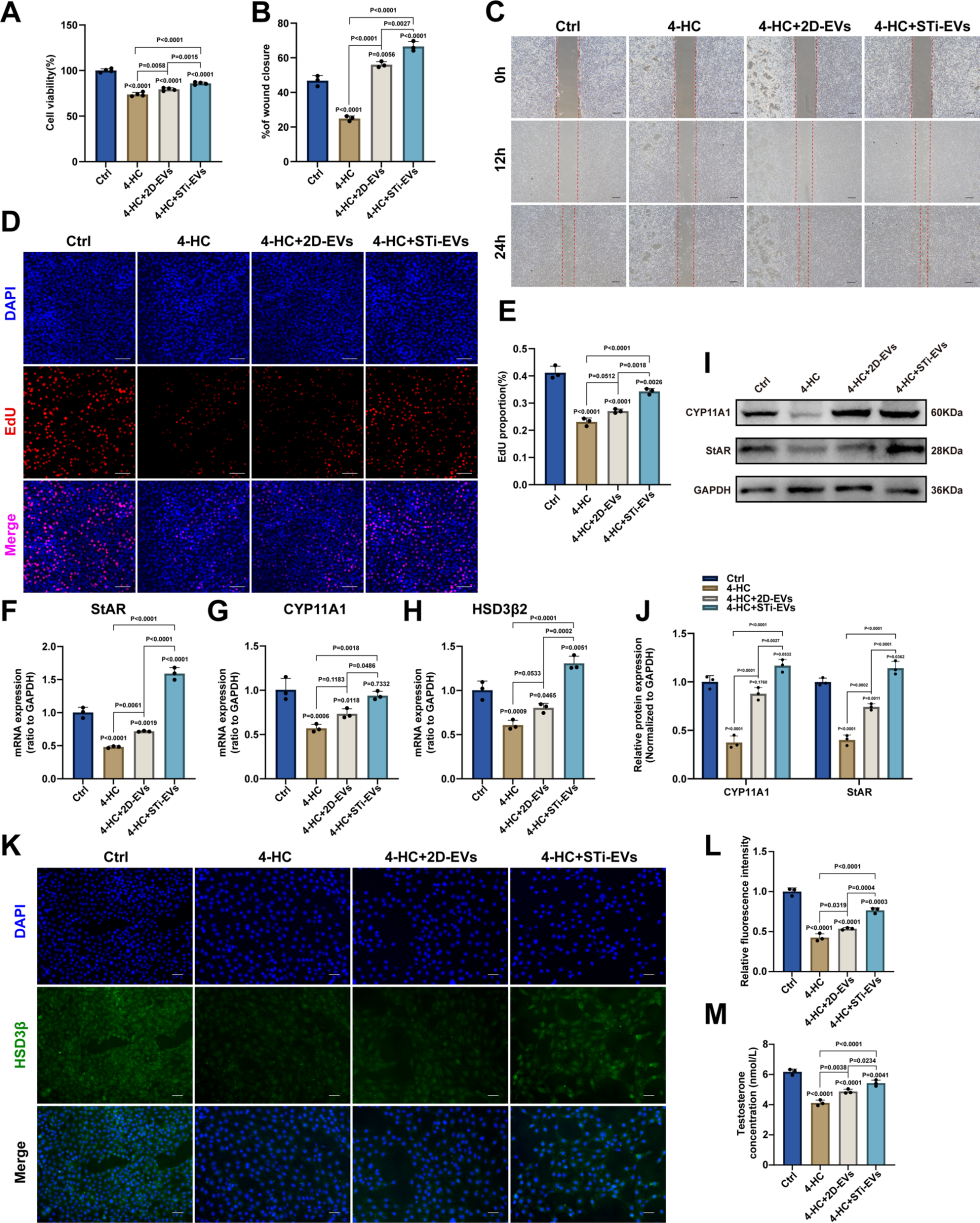
STi-EVs more effectively rescue 4-HC–induced injury and restore steroidogenesis in TM3 cells compared with 2D-EVs. (**A**) Cell viability measured by CCK-8. (**B**) Quantification of scratch wound closure. Scale bar = 250 μm. (**C**) Representative bright-field images of scratch assays at 12 h and 24 h. (**D**) Representative EdU incorporation images. Scale bar = 50 μm. **(E**) Quantification of EdU-positive cells. (**F–H**) qPCR analysis of StAR, CYP11A1, and HSD3β2 mRNA expression. (**I)** Western blot analysis of steroidogenesis-related proteins. (**J**) Densitometric quantification of western blot. (**K**) Representative immunofluorescence staining of HSD3β. Scale bar = 50 μm. (**L**) Quantification of relative HSD3β fluorescence intensity. (**M**) Testosterone concentration of TM3 cells after different treatments. Data are presented as mean ± SD. *n* = 3 per group

Using the standardized 3 µM 4-HC injury paradigm (24 h), TM3 cells were subsequently treated with 2D-EVs or STi-EVs for an additional 24 h. EV supplementation improved cell fitness across multiple readouts, with STi-EVs consistently showing a stronger restorative profile. Notably, a CCK-8 dose–response assay under the 4-HC injury model demonstrated that 1 × 10^9 particles/mL was the optimal concentration for both 2D-EVs and STi-EVs, as increasing the dose did not further enhance cell viability whereas lower doses produced only marginal changes; therefore, 1 × 10^9 particles/mL was used for subsequent experiments (Fig. S1). CCK-8 assays indicated that STi-EVs more effectively recovered cell viability compared with 2D-EVs (Fig. 4A). Scratch assays at 12 h and 24 h demonstrated enhanced wound closure in EVs-treated groups, again favoring STi-EVs (Fig. 4B–C). EdU staining further supported improved proliferative recovery following STi-EVs treatment (Fig. 4D–E). At the molecular level, qPCR and western blot analyses consistently showed that 4-HC suppressed StAR, CYP11A1, and HSD3β2 at both the mRNA and protein levels, whereas EV treatment restored these steroidogenic readouts, with a more pronounced rescue achieved by STi-EVs (Fig. 4F–J). Immunofluorescence for HSD3β corroborated recovery of steroidogenic capacity at the cellular level (Fig. 4K–L). Consistent with reinstatement of the steroidogenic program, testosterone output decreased from 6.18 ± 0.19 nmol/L (Ctrl) to 4.12 ± 0.18 nmol/L (4-HC), and was rescued to 4.88 ± 0.14 nmol/L by 2D-EVs and to 5.42 ± 0.20 nmol/L by STi-EVs (Fig. 4M).

### STi-EVs ameliorate CTX-induced testicular injury and restore endocrine output in vivo

**Fig. 5:**
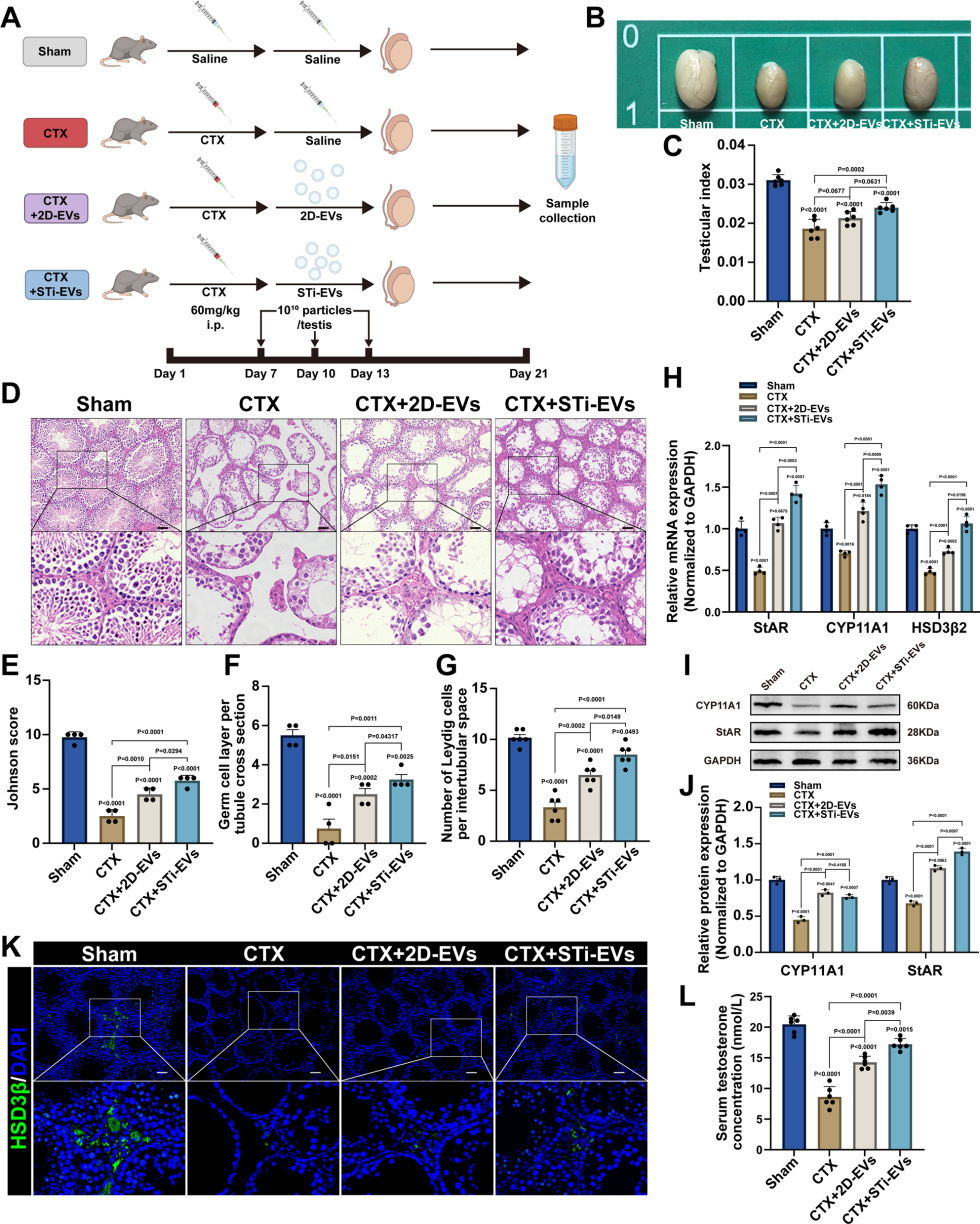
STi-EVs more effectively alleviate CTX-induced testicular injury in vivo and restore steroidogenic capacity and serum testosterone. (**A**) Diagram shows the animal experimental design. (**B**) Representative gross morphology of testes across groups. (**C**) Testis index quantification. *n* = 6 per group. (**D**) Representative hematoxylin and eosin staining of testicular sections. Scale bar = 50 μm. (**E**) Johnsen score analysis *n* = 4 per group. (**F**) Quantification of seminiferous epithelial layers. *n* = 6 per group. (**G**) Quantification of Leydig cell counts per interstitial area. *n* = 6 per group. (**H**) qPCR analysis of intratesticular steroidogenic mRNAs (StAR, CYP11A1, CYP17A1, HSD3β2). *n* = 4 per group. (**I**) Western blot analysis of CYP11A1 and StAR, with GAPDH as the loading control. (**J**) Densitometric quantification of western blot. *n* = 3 per group. (**K**) Representative immunofluorescence images of HSD3β in testicular sections. Scale bar = 50 μm. (**L**) Testosterone concentration of serum testosterone after different treatments. *n* = 6 per group. Data are presented as mean ± SD

To validate the in vivo reparative effects of STi-EVs on CTX-induced testicular injury, we established a chemotherapy-related gonadotoxicity mouse model and subsequently administered extracellular vesicles as treatment. Mice were euthanized 21 days after surgery for sample collection (Fig. 5A). Mouse body weight was monitored from day 1 to day 21 to assess systemic tolerability, and no mortality was observed during the experimental period. CTX-treated mice showed an early decrease in body weight followed by gradual recovery, whereas sham mice gained weight steadily (Fig. S3A). At day 21, all CTX-exposed groups had significantly lower body weight than the sham group (Fig. S3B). To further assess systemic safety, major organs were collected at day 21 for H&E staining. No overt histopathological abnormalities were observed in the lung, heart, liver, spleen, or kidney across groups (Fig. S4), suggesting good in vivo tolerability under the current conditions. Gross inspection revealed overt testicular atrophy after CTX exposure, whereas 2D-EVs and STi-EVs mitigated these changes, with more apparent restoration in the STi-EVs group (Fig. 5B). Consistently, the testis index was markedly reduced in the CTX group and was partially rescued by 2D-EVs and STi-EVs, with a greater improvement observed following STi-EVs compared with 2D-EVs (Fig. 5C). Histological analyses substantiated these observations. Hematoxylin and eosin staining showed that CTX disrupted seminiferous tubule organization, thinned the seminiferous epithelium, and reduced germ cell content; treatment with 2D-EVs and STi-EVs improved tubule architecture and epithelial integrity, with the STi-EVs group exhibiting the most preserved morphology and epithelial thickness (Fig. 5D). Accordingly, Johnsen scores were significantly decreased by CTX and were restored by 2D-EVs and STi-EVs, with a stronger recovery in the STi-EVs group (Fig. 5E). Quantification of seminiferous epithelial layers showed a parallel pattern, indicating that STi-EVs more effectively reinstated epithelial stratification than 2D-EVs (Fig. 5F). In the interstitial compartment, Leydig cell counts per unit area were reduced after CTX challenge and were recovered by 2D-EVs and STi-EVs, again with superior restoration in the STi-EVs group (Fig. 5G). At the molecular level, qPCR demonstrated that CTX suppressed intratesticular steroidogenic transcripts (StAR, CYP11A1 and HSD3β2), whereas treatment with 2D-EVs and STi-EVs reinstated these mRNAs, with a more robust effect in the STi-EVs group (Fig. 5H). Immunoblotting corroborated these findings, showing reduced abundance of CYP11A1 and StAR in CTX-treated testes and significant restoration following 2D-EVs and STi-EVs; densitometric analysis indicated a greater rescue by STi-EVs, with GAPDH used as the loading control for normalization (Fig. 5I–J). In addition, immunofluorescence staining of HSD3β in testicular sections showed diminished HSD3β signal after CTX exposure, which was restored by 2D-EVs and more prominently by STi-EVs (Fig. 5K). Functionally, serum testosterone fell markedly from 20.48 ± 1.37 nmol/L in Sham mice to 8.63 ± 1.69 nmol/L after CTX exposure, and was restored by 2D-EVs and STi-EVs to 14.27 ± 0.99 nmol/L and 17.22 ± 0.94 nmol/L, respectively (Fig. 5L). Collectively, these data indicate that STi-EVs confer superior in vivo efficacy, coupling histological preservation of the spermatogenic niche with enhanced recovery of Leydig cell steroidogenic capacity and systemic testosterone output.

### Transcriptomics prioritize Wnt signaling and nominate Wnt4 as a candidate axis underlying STi-EVs efficacy

**Fig. 6:**
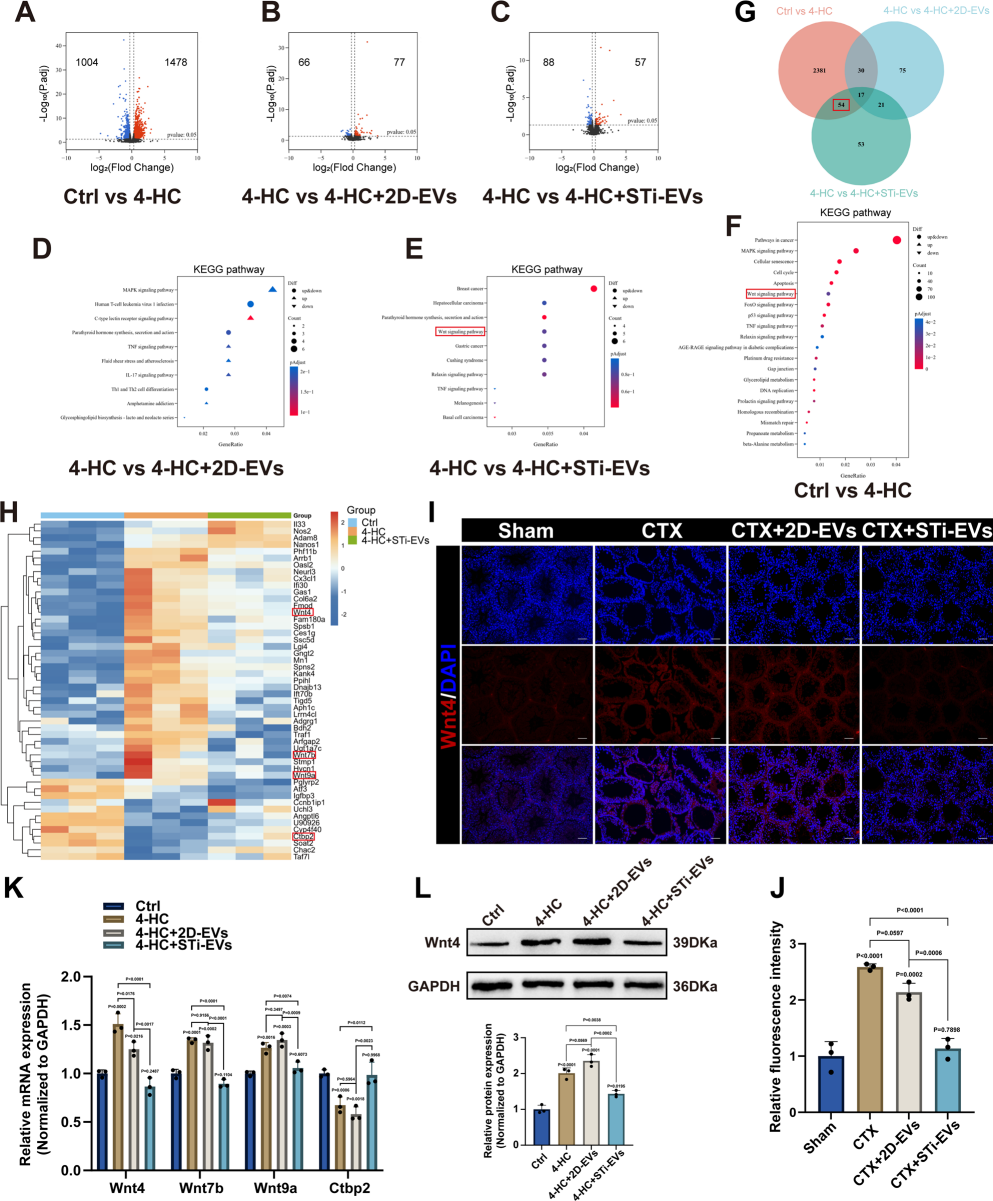
Transcriptomic prioritization and validation implicate Wnt4-associated signaling in the enhanced restorative profile conferred by STi-EVs. (**A**) Volcano plot of DEGs between Control and 4-HC. (**B**) Volcano plot of DEGs between 4-HC and 4-HC +2D-EVs. (**C**) Volcano plot of DEGs between 4-HC and 4-HC + STi-EVs. (**D**) KEGG enrichment of DEGs from 4-HC versus 4-HC +2D-EVs. (**E**) KEGG enrichment of DEGs from 4-HC versus 4-HC + STi-EVs. (**F**) KEGG enrichment of DEGs from Control versus 4-HC. (**G**) Venn diagram of TM3 cells’ DEGs after 4-HC, 2D-EVs, STi-EVs treatment. (**H**) Heatmap of the 54 candidate genes; Wnt-related genes (Wnt4, Wnt7b, Wnt9a, Ctbp2) are highlighted in the red box. (**I**) Representative immunofluorescence images of Wnt4 in testicular sections. Scale bar = 50 μm. (**J**) Quantification of relative Wnt4 immunofluorescence intensity. (**K**) qPCR validation of selected Wnt pathway–related genes. (**L**) Western blot analysis of Wnt4 and densitometric quantification across four groups. *n* = 3 per group

To elucidate molecular programs associated with EV-mediated rescue and to compare the intervention profiles of 2D-EVs and STi-EVs, we performed mRNA sequencing across four TM3 groups (Control, 4-HC, 4-HC +2D-EVs, 4-HC + STi-EVs). Volcano plots revealed extensive transcriptional reprogramming induced by 4-HC, with 1478 upregulated genes and 1004 downregulated genes relative to Control. 77 upregulated genes and 66 downregulated genes were identified between 4-HC and 4-HC +2D-EVs, whereas 57 upregulated genes and 88 downregulated genes were observed between 4-HC and 4-HC + STi-EVs (Fig. 6A–C). KEGG enrichment analyses identified a set of overlapping enriched terms between Control versus 4-HC and Control versus 4-HC + STi-EVs, which included Wnt signaling; notably, Wnt signaling was not enriched in the 4-HC versus 4-HC +2D-EVs comparison (Fig. 6D–F). Guided by this pathway level signal, we next performed a candidate-prioritization analysis aimed at identifying injury-linked genes preferentially modulated by STi-EVs rather than genes shared across both EV treatments. To this end, we intersected DEGs from Control versus 4-HC and 4-HC versus 4-HC + STi-EVs, while excluding genes also present in 4-HC versus 4-HC +2D-EVs, yielding 54 candidate genes (Fig. 6G). Heatmap visualization highlighted Wnt-associated candidates (red box), including Wnt4, Wnt7b, Wnt9a, and Ctbp2 (Fig. 6H), and qPCR validation confirmed concordant mRNA trends (Fig. 6K).

Mechanistically, canonical Wnt signaling is initiated by Wnt ligand binding to Frizzled receptors together with LRP5/6, thereby modulating β-catenin stability and TCF/LEF-dependent transcription. In the current literature, Wnt7b has been studied predominantly in tumor biology and skeletal processes, whereas Wnt9a has been mainly described outside the testis, including in skeletal development and kidney-related disorders. By contrast, Wnt4 has been implicated in gonadal differentiation and repression of steroidogenic programs, and aberrant Wnt/β-catenin activation can compromise testicular development and function. CTBP2 has been reported to constrain β-catenin–dependent transcription, thereby limiting canonical Wnt/β-catenin pathway output. Based on our transcriptomic dataset, Wnt4 mRNA increased after 4-HC exposure and decreased after STi-EVs treatment (Fig. 6H, K), nominating Wnt4 as a candidate node. We therefore validated Wnt4 in vivo: immunofluorescence staining of testicular sections indicated elevated Wnt4 signal under CTX, which shifted toward a homeostatic pattern after STi-EVs therapy, supported by quantitative analysis (Fig. 6I–J). Western blotting further confirmed significant between-group differences in Wnt4 protein abundance, aligning with the immunofluorescence findings (Fig. 6L). Collectively, integrative transcriptomic prioritization and independent validation nominate Wnt4-linked signaling as a candidate pathway associated with the superior activity of STi-EVs relative to 2D-EVs.

### STi-EVs partially reverse Wnt4 overexpression–driven suppression of steroidogenesis

**Fig. 7:**
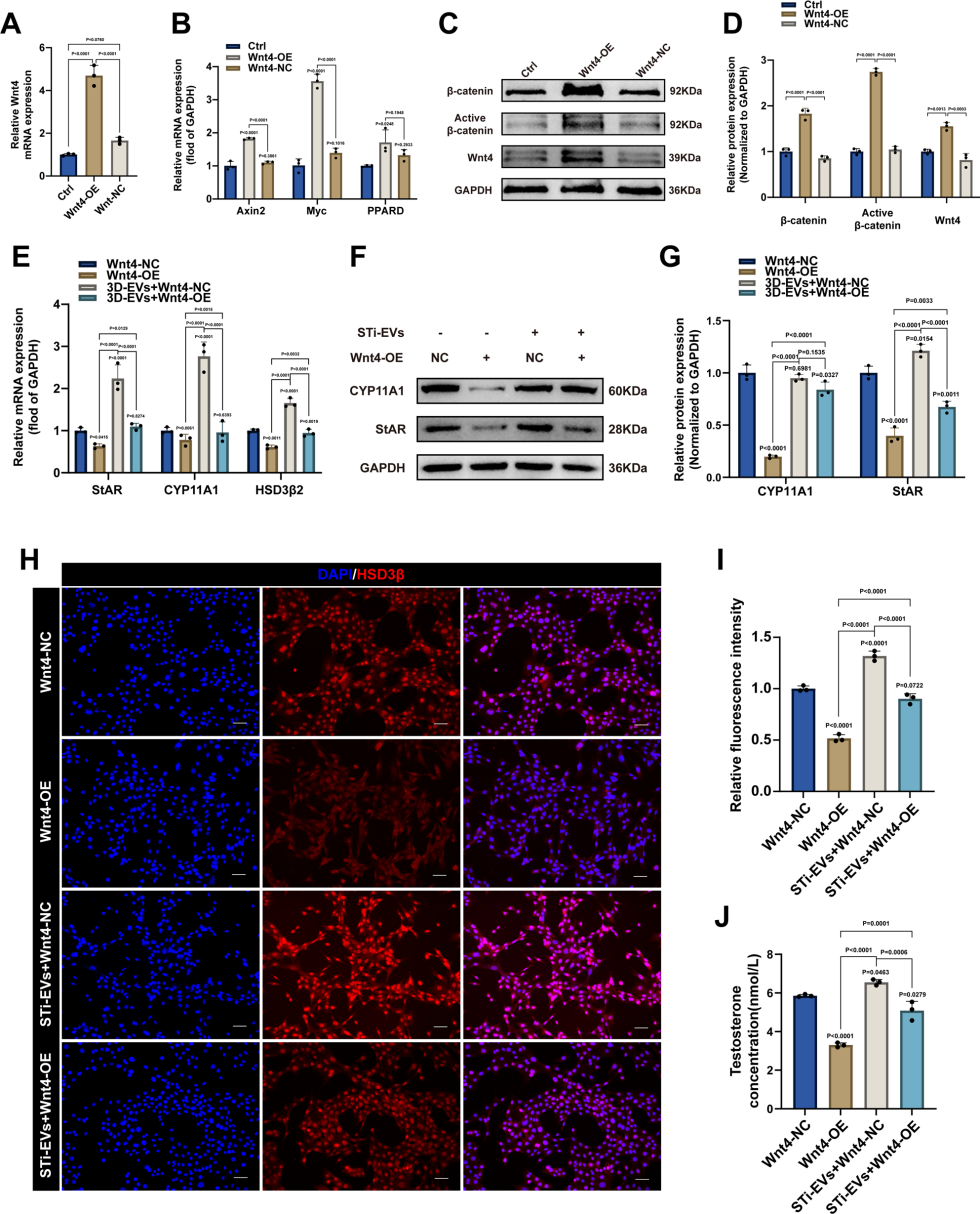
STi-EVs partially restore steroidogenic capacity under Wnt4 overexpression and mitigate Wnt/β-catenin–associated activation. (**A**) Wnt4 mRNA expression in Ctrl, empty-vector control (Wnt4-NC), and Wnt4 overexpression (Wnt4-OE) conditions. (**B**) mRNA expression of canonical Wnt/β-catenin target genes Axin2, Myc, and PPARD in Ctrl, Wnt4-NC, and Wnt4-OE groups. (**C**) Western blots of β-catenin, active β-catenin, and Wnt4 in Ctrl, Wnt4-NC, and Wnt4-OE groups. (**D**) Densitometric quantification of panel C. Ns, *P < 0.05, **P < 0.01 and ***P < 0.001; Ns, not significant (*n* = 3). (**E**) mRNA expression of StAR, CYP11A1, and HSD3β2 in Wnt4-NC, Wnt4-OE, STi-EVs + Wnt4-NC, and STi-EVs + Wnt4-OE groups. (**F**) Western blot analysis of CYP11A1 and StAR across groups. (**G**) Densitometric quantification of panel F. (**H**) Representative immunofluorescence images of HSD3β across groups. Scale bar = 50 μm. (**I**) Quantification of relative HSD3β fluorescence intensity from panel H. (**J**) Testosterone levels in culture supernatants. *n* = 3 per group

To determine whether STi-EVs can counteract the steroidogenic suppression imposed by Wnt4 overexpression, we first established a Wnt4-overexpression model in TM3 cells and verified pathway activation. In this model, Wnt4 overexpression was used as a functional perturbation to enhance Wnt4-linked canonical signaling, which is consistent with increased ligand availability and downstream β-catenin activation. qPCR confirmed robust elevation of Wnt4 mRNA in the Wnt4-OE condition (Fig. 7A), accompanied by increased expression of canonical Wnt/β-catenin target genes Axin2, Myc, while PPARD remained unchanged across groups (Fig. 7B). At the protein level, Wnt4 overexpression was associated with higher abundance of β-catenin and active β-catenin, supporting effective activation of canonical Wnt/β-catenin signaling (Fig. 7C–D). The active β-catenin/total β-catenin ratio was also increased in the Wnt4-OE group (Fig. S5A), further supporting canonical Wnt/β-catenin activation. We then focused on the restorative capacity of STi-EVs under Wnt4 overexpression. Relative to Wnt4-NC, Wnt4-OE reduced mRNA expression of key steroidogenic genes (StAR, CYP11A1, and HSD3β2). Importantly, addition of STi-EVs in the Wnt4-OE background induced a significant rebound of these transcripts, indicating that STi-EVs can partially reverse Wnt4-driven transcriptional suppression of the steroidogenic program (Fig. 7E). Consistently, immunoblotting showed that Wnt4 overexpression decreased CYP11A1 and StAR protein abundance, whereas STi-EVs shifted both proteins toward a control-like state, as supported by densitometric quantification (Fig. 7F–G). Immunofluorescence further corroborated this rescue pattern: HSD3β signal was diminished by Wnt4 overexpression and was partially restored following STi-EVs treatment, with quantitative intensity analysis aligning with representative images (Fig. 7H–I). Functionally, testosterone measurements in culture supernatants showed that Wnt4 overexpression reduced testosterone from 5.856 ± 0.07 nmol/L in Wnt4-NC to 3.30 ± 0.12 nmol/L in Wnt4-OE. Upon STi-EVs treatment, testosterone increased to 6.55 ± 0.15 nmol/L in the Wnt4-NC background and was restored to 5.09 ± 0.48 nmol/L even under Wnt4 overexpression (Fig. 7J). Collectively, these results demonstrate that STi-EVs partially restore steroidogenic gene expression, protein abundance, and testosterone output in the setting of Wnt4 overexpression, implicating an antagonistic effect on Wnt4/β-catenin–associated inhibitory signaling.

### Wnt4 silencing dampens β-catenin signaling and rescues steroidogenesis under 4-HC stress

**Fig. 8:**
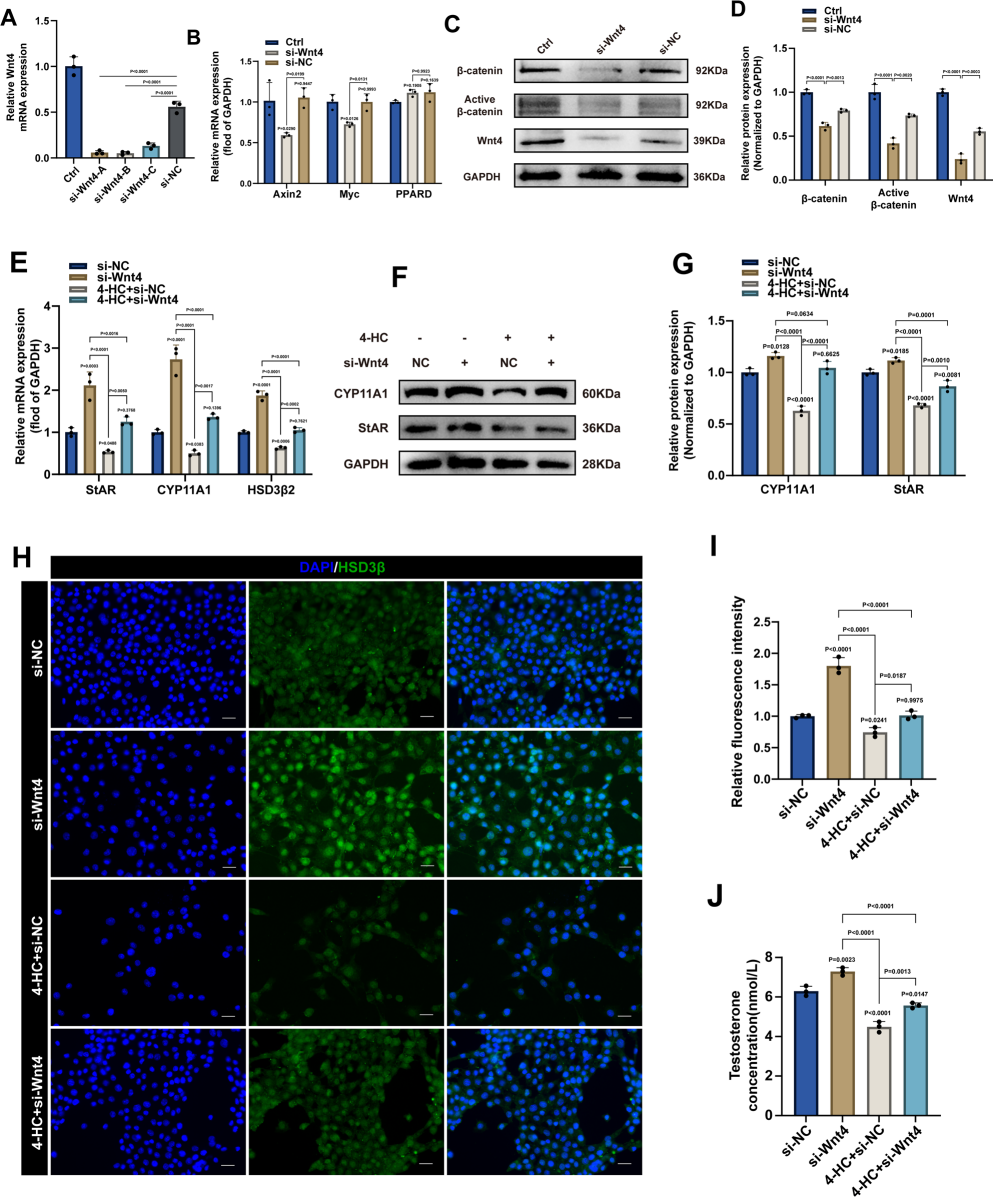
Wnt4 silencing dampens Wnt/β-catenin signaling and partially rescues 4-HC–induced steroidogenic deficits and testosterone output. (**A**) Wnt4 mRNA expression after transfection with si-Wnt4-a/b/c, together with si-NC and Ctrl; si-Wnt4-b was selected for subsequent experiments based on knockdown efficiency. (**B**) mRNA expression of canonical Wnt/β-catenin target genes Axin2, Myc, and PPARD in Ctrl, si-NC, and si-Wnt4 groups. (**C**) Western blot of β-catenin, active β-catenin, and Wnt4 in Ctrl, si-NC, and si-Wnt4 groups. (**D**) Densitometric quantification of panel C. Ns, *P < 0.05, **P < 0.01 and ***P < 0.001; Ns, not significant (*n* = 3). (**E**) mRNA expression of StAR, CYP11A1, and HSD3β2 in si-NC, si-Wnt4, 4-HC + si-NC, and 4-HC + si-Wnt4 groups. (**F**) Western blot analysis of CYP11A1 and StAR across groups. (**G**) Densitometric quantification of panel F. (**H**) Representative immunofluorescence images of HSD3β across groups. Scale bar = 50 μm. (**I**) Quantification of relative HSD3β fluorescence intensity from panel H. (**J**) Testosterone levels in culture supernatants. *n* = 3 per group

To further test the functional relevance of Wnt4, TM3 cells were transfected with three Wnt4-targeting siRNAs (si-Wnt4-a/b/c), together with si-NC and Ctrl. qPCR screening identified si-Wnt4-b as the most efficient sequence and it was selected for subsequent experiments (Fig. 8A). To further support sequence robustness, we additionally examined downstream canonical Wnt/β-catenin readouts across all three Wnt4-targeting siRNAs and found that si-Wnt4-a/b/c each reduced Axin2 and Myc expression (Fig. S6). In pathway readouts, Wnt4 silencing decreased Axin2 and Myc mRNA expression, whereas PPARD showed no difference across groups (Fig. 8B). Western blotting confirmed reduced Wnt4 protein and decreased β-catenin and active β-catenin, consistent with attenuated pathway output (Fig. 8C–D). Wnt4 silencing reduced the active β-catenin/total β-catenin ratio (Fig. S5B), consistent with attenuated canonical Wnt/β-catenin signaling. Functionally, Wnt4 silencing increased StAR, CYP11A1, and HSD3β2 expression and partially reversed their suppression under 4-HC challenge (Fig. 8E). Consistently, western blot analysis demonstrated increased CYP11A1 and StAR protein abundance with si-Wnt4 and a partial reversal of the 4-HC–associated protein suppression (Fig. 8F–G). Immunofluorescence further confirmed recovery of the steroidogenic phenotype, with reduced HSD3β signal in 4-HC + si-NC cells and increased signal in 4-HC + si-Wnt4 cells (Fig. 8H–I). Functionally, testosterone levels in culture supernatants were 6.30 ± 0.25 nmol/L in the si-NC group and increased to 7.29 ± 0.20 nmol/L after Wnt4 knockdown; 4-HC reduced testosterone to 4.50 ± 0.27 nmol/L, whereas Wnt4 knockdown partially restored output to 5.57 ± 0.13 nmol/L under 4-HC exposure (Fig. 8J). Collectively, these data indicate that Wnt4 silencing dampens β-catenin pathway output and strengthens the steroidogenic program, thereby partially restoring testosterone biosynthesis under 4-HC challenge.

Taken together, the gain-of-function and loss-of-function analyses converge on a reciprocal pattern: Wnt4 upregulation activates β-catenin–associated signaling and suppresses steroidogenic capacity, whereas Wnt4 silencing drives an opposing molecular and functional shift that partially restores testosterone biosynthesis. Collectively, these mirror-image phenotypes position the Wnt4/β-catenin axis as a central regulatory node and provide final mechanistic support for a model in which STi-EVs restore Leydig-like steroidogenesis, at least in part, through repression of Wnt4-linked signaling.

To further test whether β-catenin activity is functionally involved in STi-EV-mediated steroidogenic rescue, we performed a pharmacological pathway-intervention experiment using CHIR-99,021, a β-catenin stabilizer. CCK-8 analysis showed a fitted IC₅₀ of 6.037 µM in TM3 cells (Fig. S9A). We then compared 1 µM and 3 µM CHIR-99,021 and found that 3 µM more strongly induced the canonical β-catenin target genes Axin2 and Myc without marked cytotoxicity; therefore, 3 µM was selected for subsequent experiments (Fig. S9B).

Under 4-HC injury, STi-EVs restored the expression of steroidogenesis-related genes and testosterone production. However, co-treatment with CHIR-99,021 significantly attenuated the STi-EV-mediated recovery of StAR, CYP11A1, and HSD3β2 mRNA expression (Fig. S9C). Consistently, Western blotting showed that CHIR-99,021 increased β-catenin pathway activation and weakened the restoration of StAR protein induced by STi-EVs (Fig. S9D–F). ELISA further confirmed that CHIR-99,021 significantly reduced the testosterone recovery achieved by STi-EVs (Fig. S9G). These data indicate that pharmacological stabilization of β-catenin partially blocks STi-EV-mediated steroidogenic rescue, supporting the functional involvement of β-catenin restraint in the protective effect of STi-EVs.

## Discussion

This work links chemotherapy-associated Leydig cell endocrine injury to a manufacturable extracellular vesicle strategy by establishing a seminiferous tubule–inspired coaxial bioprinting encapsulation culture for scalable hUMSC EV production (termed STi-EVs) and by identifying a pathway-modulation mechanism associated with functional rescue. Across a 4-HC–injured TM3 model and a CTX mouse model, STi-EVs consistently outperformed conventional 2D-EVs in restoring cellular fitness, re-engaging core steroidogenic nodes, and improving testosterone output, while alleviating histopathology and rescuing serum testosterone in vivo. Mechanistically, transcriptome-guided prioritization integrated with orthogonal validation, Wnt4 gain- and loss-of-function experiments, and β-catenin stabilization experiments supports Wnt4/β-catenin signaling modulation as a functional contributor to the superior reparative activity of STi-EVs.

An additional point raised by the basal-treatment experiment is whether the steroidogenic-promoting effect of STi-EVs in healthy TM3 cells is connected to the same Wnt4/β-catenin-centered mechanism observed under 4-HC injury. We therefore further examined Wnt4/β-catenin signaling in healthy TM3 cells treated with 2D-EVs or STi-EVs. Consistent with the increase in steroidogenic gene expression and testosterone output, STi-EVs significantly reduced Wnt4, total β-catenin, and active β-catenin protein levels, as well as the active β-catenin/total β-catenin ratio. In parallel, STi-EVs decreased the mRNA expression of Wnt4 and the canonical β-catenin target genes Axin2 and Myc, whereas 2D-EVs showed little or no effect on these basal Wnt-related readouts (Fig. S2). These findings suggest that STi-EVs can suppress basal Wnt4/β-catenin signaling in Leydig-like TM3 cells, which may contribute to their ability to enhance steroidogenic activity even in the absence of overt 4-HC injury.

Nevertheless, we interpret this basal effect cautiously. Healthy TM3 cells are not equivalent to primary Leydig cells under physiological conditions, and basal steroidogenesis may be regulated by multiple pathways beyond Wnt4/β-catenin signaling. Therefore, although the expanded Fig. S2 supports a mechanistic connection between basal STi-EV activity and reduced Wnt4/β-catenin signaling, future studies in primary Leydig cells and in vivo basal-treatment models will be needed to determine whether this effect reflects a broader physiological steroidogenic modulation or a TM3 cell-specific response.

Clinically, CTX remains a prototypical alkylating agent used in oncologic and immunologic indications; as survival improves, late endocrine sequelae have become increasingly relevant.[33] In male survivors of childhood cancer, direct assessments indicate that Leydig cell dysfunction and Leydig cell failure are clinically meaningful entities, and risk increases with alkylating exposure—most notably with cyclophosphamide equivalent doses at or above 4000 mg/m², which emerged as an independent risk factor for Leydig cell failure.[17] While testosterone replacement can normalize circulating androgen levels, it does not rebuild intrinsic steroidogenic capacity; moreover, major guidelines recommend against initiating testosterone therapy in men planning fertility in the near term, underscoring the need for mechanism-guided strategies that restore endogenous steroidogenesis.[34, 35].

Although the CTX regimen used here generated a reproducible gonadotoxic phenotype and allowed us to evaluate STi-EV-mediated endocrine rescue, it should be interpreted as a preclinical proof-of-concept injury model rather than a direct equivalent of clinical cyclophosphamide exposure. Clinically, male gonadal injury after cyclophosphamide or other alkylating agents is strongly influenced by cumulative exposure, treatment context, developmental stage, and recovery interval.[36] Higher cumulative alkylating-agent exposure has been associated with impaired spermatogenesis, infertility risk, and, in some settings, Leydig cell dysfunction, while recovery may be variable and can occur over prolonged timeframes.[37] Therefore, the 60 mg/kg ×7 days CTX regimen used in this study is best viewed as an established murine injury protocol that produces a robust and time-bounded testicular damage model for therapeutic evaluation, rather than as a direct clinical dose simulation. Future studies using multiple CTX dose levels, longer recovery windows, and endocrine/fertility follow-up will be needed to better align preclinical efficacy testing with clinically heterogeneous gonadotoxicity trajectories.

In this study, we adopted a fixed dosing regimen primarily to support standardized comparisons between 2D-EVs and STi-EVs while interrogating endocrine rescue within a defined post-chemotherapy window. For in vitro experiments, we performed an additional CCK-8 dose–response assay under the 4-HC injury model (Fig. S1), which supported 1 × 10^9 particles/mL as a practical working concentration for both 2D-EVs and STi-EVs, because increasing the dose above this level did not further improve viability whereas lower doses produced only marginal effects; accordingly, this concentration was used throughout subsequent in vitro studies. For in vivo studies, we applied EVs by intratesticular injection at 1 × 10^10 particles per testis, administered three times (days 7, 10, and 13) following the 7-day CTX regimen, and evaluated outcomes at day 21. This repeated-dosing design was selected to sustain local exposure during the early recovery period and to maintain identical dosing parameters across EV groups. We acknowledge, however, that a systematic in vivo dose–response assessment and longer-term durability evaluation were beyond the scope of the present work. Future studies will therefore define the minimal effective dose, optimize dosing intervals and total dosing burden, and assess the persistence of endocrine recovery and safety over extended follow-up—an important step for translating manufacturable EV supply into practical repeated-dosing regimens for testis endocrine indications.

Although we used intratesticular injection to maximize local exposure and to test endocrine rescue directly in the target organ, we acknowledge that this route is invasive and may limit immediate clinical applicability as a routine administration strategy. Therefore, the present in vivo delivery should be interpreted primarily as a proof-of-concept approach to evaluate organ-level efficacy under controlled exposure conditions rather than as a finalized clinical regimen. Future translation will require careful assessment of delivery feasibility and safety, and may benefit from alternative administration strategies. Recent work highlights that the therapeutic efficacy of EVs depends strongly on tissue retention and uptake, motivating approaches for tissue-specific delivery and engineered EV targeting after systemic administration.[38] In parallel, local delivery platforms such as injectable hydrogels have been proposed to prolong EV retention and enable sustained release, potentially reducing injection frequency while maintaining effective exposure.[39] Finally, delivery to the testis is constrained by the blood–testis barrier, yet emerging evidence indicates that testicular extracellular vesicles can traverse this barrier and mediate inter-compartment communication, supporting the feasibility of EV-based strategies for testis-targeted delivery.[40, 41] Taken together, clinically feasible delivery and sustained exposure will be essential to translate manufacturable STi-EVs.

Notably, clinical MSC-EV dosing remains heterogeneous, with studies reporting dose units variably as protein mass, particle number, or producer-cell equivalents and without a consensus potency-linked dosing framework across indications and routes.[42, 43] As one example, a randomized, placebo-controlled dosing clinical trial of a BM-MSC-derived EV product (ExoFlo) administered 10 mL or 15 mL ExoFlo on days 1 and 4 by infusion, illustrating that clinical regimens may be defined by dose volume and repeated administration rather than particle number alone.[44] In parallel, reviews of exosome biodistribution and pharmacokinetics emphasize that delivery and clearance constraints can limit exposure duration, which is particularly relevant for repeated-dosing indications and further amplifies the need for batch-consistent manufacturing and standardized release criteria.[45] Within this landscape, the STi-EVs framework couples tubule-like geometric confinement with operational practicality (continuous harvesting, reduced volumes, compatible downstream integration), improving the feasibility of batch-consistent EV supply for endocrine-focused testis indications, while also highlighting the remaining translational bottlenecks. Specifically, downstream workflows compatible with GMP (Good Manufacturing Practice–compliant) production, lot-to-lot comparability, and potency-linked release assays remain necessary to translate a scalable upstream supply into clinically actionable repeated-dosing regimens.[43] We acknowledge that ultracentrifugation produces EV–enriched fraction and may co-isolate non-vesicular materials; therefore, higher-purity workflows (e.g., SEC or density gradients) may be considered in future studies.

A parallel translational bottleneck is manufacturability, particularly for indications that may require repeated dosing.[46] At the tissue-architecture level, the testis is dominated by seminiferous tubules, which constitute most of the organ’s parenchyma and define a stable tubular microenvironment where spermatogenesis is organized and compartmentalized. This tubular organization is functionally coupled to barrier physiology: Sertoli–Sertoli tight junction complexes at the seminiferous epithelium form the blood–testis barrier, creating discrete basal and adluminal compartments and reinforcing the notion that three-dimensional confinement and controlled transport are intrinsic design features of the native testis.[47] Motivated by this strictly structural rationale, this study implemented coaxial bioprinting to generate tubular core–shell hydrogel constructs that impose stable 3D confinement while remaining permissive for mass transport, thereby sustaining high viability and secretory activity under high-density culture and enabling continuous recovery of conditioned medium in compact volumes. Consistent with this engineering logic, a related coaxial hollow-microfiber platform has been reported to markedly improve EV manufacturability when quantified using standardized per-dish outputs (particles/petri dish and µg EV protein/petri dish) and particle-to-protein ratios; notably, EV particle yield increased from ~2.5 × 10^8^ to ~2.5 × 10^11^ particles per dish (≈ 1000-fold), EV protein increased from ~4.4 to ~203 µg per dish (≈ 46-fold), and the particle-to-protein ratio improved by ≈ 14-fold, while also reducing space, labor, time, and cost.[14, 48] This design also provides a foundation for further optimization of production-related parameters, including extrusion-associated shear, matrix-dependent transport characteristics, construct uniformity, yield per volume or time, cost-relevant metrics, batch-to-batch reproducibility, and head-to-head scalability versus other platforms. Continued refinement along these directions may further strengthen the utility of this system as a more controllable, reproducible, and scalable EV manufacturing strategy.

Beyond coaxial printing, hollow-fiber bioreactors represent another major route for continuous and homogeneous MSC-EV production, and are frequently discussed as clinically compatible manufacturing candidates.[49] Together, these advances indicate that upstream architecture is becoming a central determinant of EV scalability; within this landscape, the STi-EV framework couples tubule-like geometric confinement with operational practicality (continuous harvesting, reduced volumes, compatible downstream integration), improving the feasibility of batch-consistent EV supply for endocrine-focused testis indications.

Notably, 3D culture may confer advantages beyond “more EV.” Multiple studies show that EV produced in 3D versus 2D conditions can differ in secretion dynamics and molecular cargo, including small-RNA content that more closely reflects in vivo-like signatures.[50] Other work further suggests that substantial proteomic differences can arise between 2D- and 3D-derived EV even when morphology, size, and classical EV markers appear broadly comparable.[51] In this context, the superior activity of STi-EVs in rescuing Leydig-like steroidogenesis should not be reduced to a yield effect alone; rather, it supports a model in which the 3D encapsulation microenvironment helps shape an evaluable functional phenotype, thereby linking scalability with potency. Guided by the Wnt4/β-catenin-centered mechanism identified in this study, we performed a targeted comparison of Wnt-related proteins in MSCs, 2D-EVs, and STi-EVs. Wnt4 and DKK1 were barely detectable in EV preparations, whereas SFRP1 was detectable and appeared enriched in STi-EVs. To improve the interpretability of this cargo comparison, we further normalized SFRP1 abundance to the EV-associated marker TSG101 and performed densitometric quantification across biological replicates. This analysis showed significantly higher TSG101-normalized SFRP1 abundance in STi-EVs than in 2D-EVs (Fig. S7), suggesting that STi-EVs may contain higher levels of EV-associated Wnt-modulatory cargo. Because SFRP1 is a secreted Wnt antagonist, this result provides a plausible cargo-level clue for the stronger Wnt4/β-catenin-modulatory activity of STi-EVs.[25, 52, 53] Nevertheless, we interpret this finding cautiously, as SFRP1 depletion/neutralization, recombinant SFRP1 gain-of-function assays, and broader cargo profiling will be required to determine whether SFRP1 is functionally necessary or sufficient for STi-EV-mediated steroidogenic rescue.

Mechanistically, canonical Wnt/β-catenin signaling is typically initiated by Wnt ligands engaging Frizzled receptors and the LRP5/6 co-receptors, which suppresses the Axin/APC/GSK3β destruction complex, stabilizes β-catenin, and enables nuclear TCF/LEF-dependent transcriptional programs.[24] In gonadal biology, Wnt4 is widely positioned as a pro-ovarian cue that is actively restrained during testis fate acquisition.[28, 54] Consistent with an endocrine-relevant role of this axis, an ex vivo human fetal testis model demonstrated that promotion of Wnt4/β-catenin signaling markedly compromises Leydig cell steroidogenic function, with reduced secretion of testosterone, androstenedione, and INSL3, linking pathway activation to impaired androgen output.[27] Placed into an injury–repair context, the present dataset supports Wnt4 as a tractable node: alkylating stress is accompanied by Wnt4 induction and heightened β-catenin output, whereas STi-EVs shift the pathway toward baseline and functional Wnt4 gain- and loss-of-function perturbations align directionally with restoration of the steroidogenic program, strengthening the causal plausibility of a Wnt4/β-catenin–centered repair axis. Although concordant pathway suppression across multiple independent siRNAs supports sequence robustness, more comprehensive off-target evaluation would be valuable in future studies.

Given the context-dependent complexity of Wnt signaling, we interpret our findings as most consistent with a canonical Wnt4/β-catenin–centered contribution to STi-EVs–mediated rescue under the tested conditions. However, Wnt signaling comprises multiple ligands and non-canonical branches that are often regulated at post-translational levels (e.g., PCP/JNK or Ca²⁺-dependent modules) and may not be fully reflected by the canonical readouts emphasized here. Moreover, STi-EVs treatment in our models results in functional recovery rather than complete normalization, suggesting that additional mechanisms may also be involved. We acknowledge that chemotherapy-associated toxicity may involve not only cell death but also senescence-related phenotypes. However, in our transcriptomic dataset, cell senescence was not identified as an enriched KEGG pathway in the 4-HC versus 4-HC + STi-EVs comparison. The present work focused on Wnt4/β-catenin–linked mechanisms that were preferentially associated with the restorative effect of STi-EVs, whereas the potential contribution of senescence- and SASP-related programs warrants dedicated investigation in future studies.

A further limitation of this study is the reliance on TM3 cells for the in vitro mechanistic experiments. TM3 cells are a mouse Leydig cell line derived from Leydig cell-enriched preparations from immature mouse testes and retain several Leydig-like features, including responsiveness to luteinizing hormone and the ability to metabolize cholesterol in the presence of LH. TM3 cells have been widely used for mechanistic studies of chemical-induced reproductive toxicity, however, TM3 cells are still an immortalized/proliferative Leydig-like model and may not fully recapitulate the steroidogenic capacity, maturation state, or signaling regulation of adult primary Leydig cells in the native testicular microenvironment.[55] Therefore, although TM3 cells provided a tractable system for transcriptomic screening, Wnt4 gain- and loss-of-function experiments, and β-catenin pharmacological intervention, the mechanistic conclusions should be interpreted as Leydig-like cell-line evidence rather than definitive proof in primary Leydig cells. The concordant in vivo findings in the CTX mouse model support the physiological relevance of STi-EV-mediated endocrine recovery, but future studies using isolated primary Leydig cells, ex vivo testicular tissue, and lineage-resolved in vivo analyses will be important to confirm whether Wnt4/β-catenin modulation operates similarly in primary Leydig cells within the native testicular niche.

We also acknowledge that the present study uses a cross-species design, with human hUMSC-derived EVs tested in murine recipient systems. While prior studies support the functional plausibility of human UCMSC-derived EV activity in murine reproductive contexts and more broadly recognize EVs as effective mediators of intercellular communication, the precise cargo–target relationships between human EV contents and murine recipient transcripts were not resolved here and warrant direct investigation in future work.[56–58] Therefore, we do not exclude broader pathway modulation beyond Wnt4 and will pursue future studies incorporating non-canonical signaling assays, expanded pathway profiling, and direct cargo–target validation to further delineate the full spectrum of STi-EVs actions.

To help integrate these considerations into a coherent mechanistic framework, we outline a working model that links EV uptake to pathway modulation and downstream steroidogenic recovery. We propose a working model with three layers. First, both EV types are internalized, establishing exposure of recipient Leydig cells to EV-associated signals. Second, qualitative differences in EV surface composition and cargo may bias receptor-proximal regulation—either by altering the effective availability of Wnt receptor complexes (FZD/LRP) at the plasma membrane, or by influencing endocytic routing (clathrin/caveolin-associated uptake, endosomal sorting) that can shape signaling outcomes. Third, these upstream differences would translate into distinct nuclear signaling dynamics, with STi-EVs leading to a reduced and/or shorter-lived β-catenin transcriptional output (e.g., Axin2/Myc), which in turn favors recovery of the steroidogenic gene network and testosterone synthesis. While this model is consistent with our observed uptake and Wnt4/β-catenin readouts, direct testing of receptor expression, trafficking routes, and β-catenin nuclear kinetics will require dedicated future studies.

In summary, this study integrates a structurally inspired, scalable EV manufacturing paradigm with a mechanistically supported Wnt4/β-catenin axis, providing a translationally relevant, cell-free framework to repair alkylating agent–associated Leydig cell steroidogenic failure.

## Supplementary Information

Supplementary Material 1.

## Data Availability

No datasets were generated or analysed during the current study.
