# Supplementary material for: Seminiferous tubule–inspired coaxial bioprinting–derived extracellular vesicles restore Leydig cell steroidogenesis through modulation of Wnt4/β-catenin signaling
Source: J Nanobiotechnology. 2026 May 21;24:670. doi: 10.1186/s12951-026-04574-0 (PMC13378318; doi:10.1186/s12951-026-04574-0)
Supplement: Supplementary file 1 — Supplementary Material 1. [file 12951_2026_4574_MOESM1_ESM.docx]

**Seminiferous tubule–inspired coaxial bioprinting–derived extracellular vesicles restore Leydig cell steroidogenesis through modulation of Wnt4/β-catenin signaling**

*Jialin Wu^1,2,†^, Jianwei Chen^3,4,†^, Yiran Tao^1,2,†^, Qiang Guo^1,2^, Jiayu Huang^1,2^, Yi Zhang^5^, Tianyou Zhang^1,2^, Zijun Mo^1,2^, Dejuan Wang^1,2,^*, Tao Xu^3,4,^*, Jianguang Qiu^1,2,^**

^1^Department of Urology, The Sixth Affiliated Hospital, Sun Yat-sen University, No 26 Yuancun Erheng Road, Guangzhou, 510655, China

^2^Biomedical Innovation Center, The Sixth Affiliated Hospital, Sun Yat-sen University, Guangzhou, 510655, China

^3^Research and Development Department(R&D)， Qingyuanzhixin（shenzhen） Biotechnology Co., LTD

^4^Center for Bio-intelligent Manufacturing and Living Matter Bioprinting, Research Institute of Tsinghua University in Shenzhen, Tsinghua University, Shenzhen 518057, China

^5^Department of Research and Development, Huaqing Zhimei (Shenzhen) Biotechnology Co., Ltd., Shenzhen, Guangdong Province 518107, China

*Corresponding author:

Jianguang Qiu - [qiujg@mail.sysu.edu.cn](mailto:qiujg@mail.sysu.edu.cn)

Tao Xu - [xut@tsinghua-sz.org](mailto:xut@tsinghua-sz.org)

Dejuan Wang - [wangdej@mail.sysu.edu.cn](mailto:wangdej@mail.sysu.edu.cn)

†These authors contributed equally to this work

Supplementary Table 1. Primers for Quantitative Real-time PCR.

| Gene | Forward Primer (5’ – 3’) | Reverse Primer (5’ – 3’) |
| --- | --- | --- |
| Mus-StAR | CCGGAGCAGAGTGGTGTCA | CAGTGGATGAAGCACCATGC |
| Mus-CYP11A1 | CACTGAGACTCCACCCCATC | GGCAAAGCTAGCCACCTGTA |
| Mus-HSD3β2 | AGCTCTGGACAAAGTATTCCGA | GCCTCCAATAGGTTCTGGGT |
| Mus-GAPDH | GTCTTCACTACCATGGAGAAGG | TCATGGATGACCTTGGCCAG |
| Mus-Wnt4 | CGAGCAATTGGCTGTACCTG | GGGAGTCCAGTGTGGAACAG |
| Mus-Wnt7b | ATCCTACTATGTGTCGCCCG | CACGGATGACAATGCTCTGTAAG |
| Mus-Wnt9a | GGTGGGCAAGCACCTAAAAC | GTACAAGCTCTGGTGTTCGGG |
| Mus-Ctbp2 | GTCCCTACTGAGAACAGCACG | CTCAGCAGGGCCAAAAACTC |
| Mus-Axin2 | ATGAGTAGCGCCGTGTTAGTG | GGGCATAGGTTTGGTGGACT |
| Mus-Myc | GTTGGAAACCCCGCAGACAG | ATAGGGCTGTACGGAGTCGT |
| Mus-PPARD | GCAGCCTCAACATGGAATGTC | GAGCTTCATGCGGATTGTCC |

Supplementary Table 2. Si-Wnt4 sequence.

| Gene | Si-Wnt4 sequence |
| --- | --- |
| Si-Wnt4-A | F: 5’-ACGUGCAACAAGACAUCUAAATT-3’  R: 3’-UUUAGAUGUCUUGUUGCACGUTT-5’ |
| Si-Wnt4-B | F: 5’-AGACGUGCGAGAAACUCAAAGTT-3’  R: 3’-CUUUGAGUUUCUCGCACGUCUTT-5’ |
| Si-Wnt4-C | F: 5’-ACGCGCUAAAGGAGAAGUUUGTT-3’  R: 3’-CAAACUUCUCCUUUAGCGCGUTT-5’ |
| Si-NC | F: 5’-UUCUCCGAACGUGUCACGUTT-3’  R: 3’-TAAGAGGCUUGCACAGUGCA-5’ |


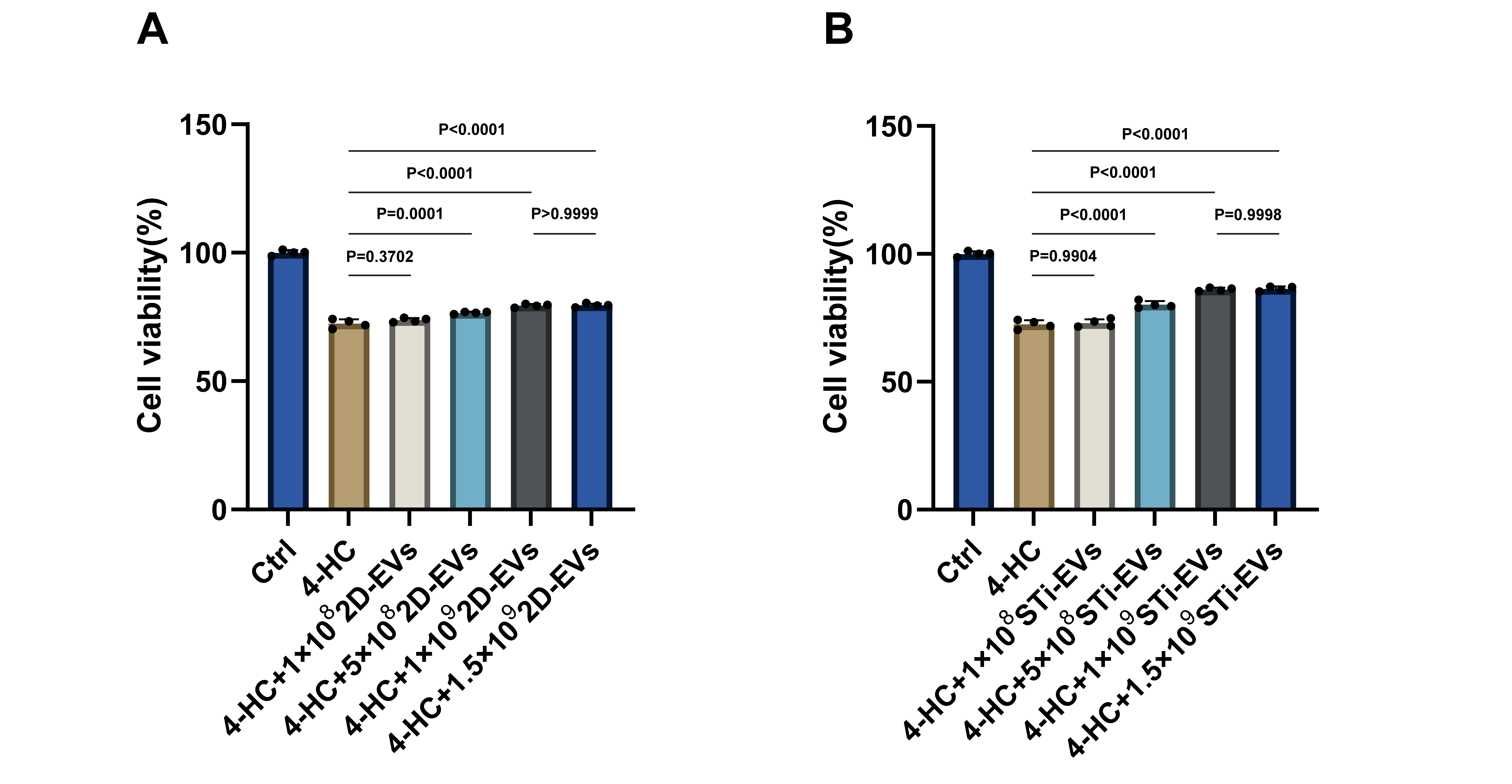


**Figure S1. CCK-8 dose screening of 2D-EVs and STi-EVs under 4-HC injury in TM3 cells.** TM3 cells were injured with 3 μM 4-HC for 24 h and then treated with increasing doses of 2D-EVs or STi-EVs for 24 h. Cell viability measured by CCK-8.


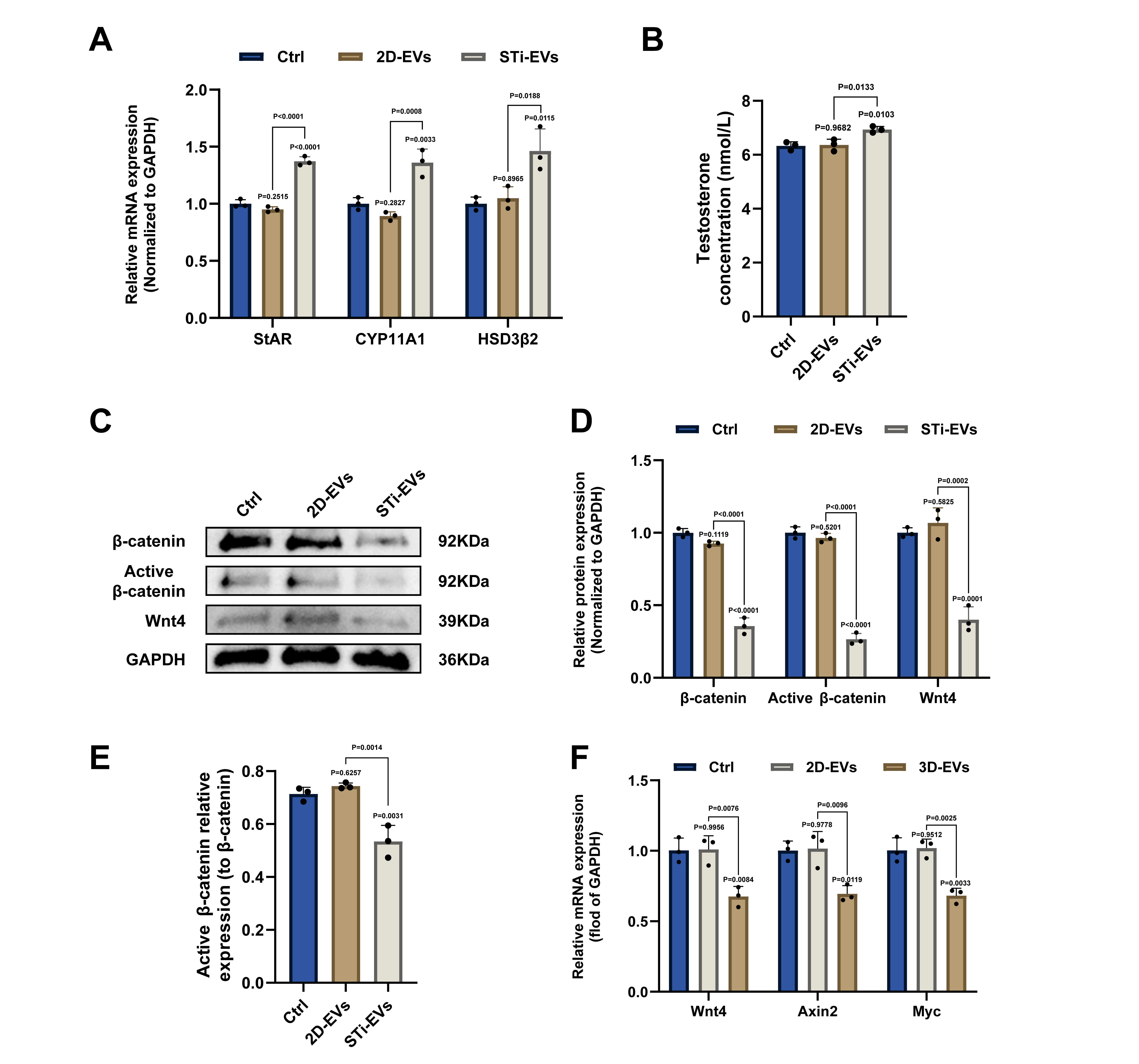


**Figure S2. Effects of 2D-EVs and STi-EVs on steroidogenic function in healthy TM3 cells.** (A) qPCR analysis of steroidogenesis-related genes in healthy TM3 cells treated with 2D-EVs or STi-EVs. (B) Testosterone concentration in the culture supernatant of healthy TM3 cells after treatment with 2D-EVs or STi-EVs. (C) Representative Western blot images showing β-catenin, active β-catenin, and Wnt4 protein expression in healthy TM3 cells after the indicated treatments. (D) Densitometric quantification of β-catenin, active β-catenin, and Wnt4 protein expression normalized to GAPDH. (E) Same-lane ratio analysis of active β-catenin relative to total β-catenin. (F) qPCR analysis of Wnt4 and canonical β-catenin target genes Axin2 and Myc in healthy TM3 cells after the indicated treatments. n = 3 per group.


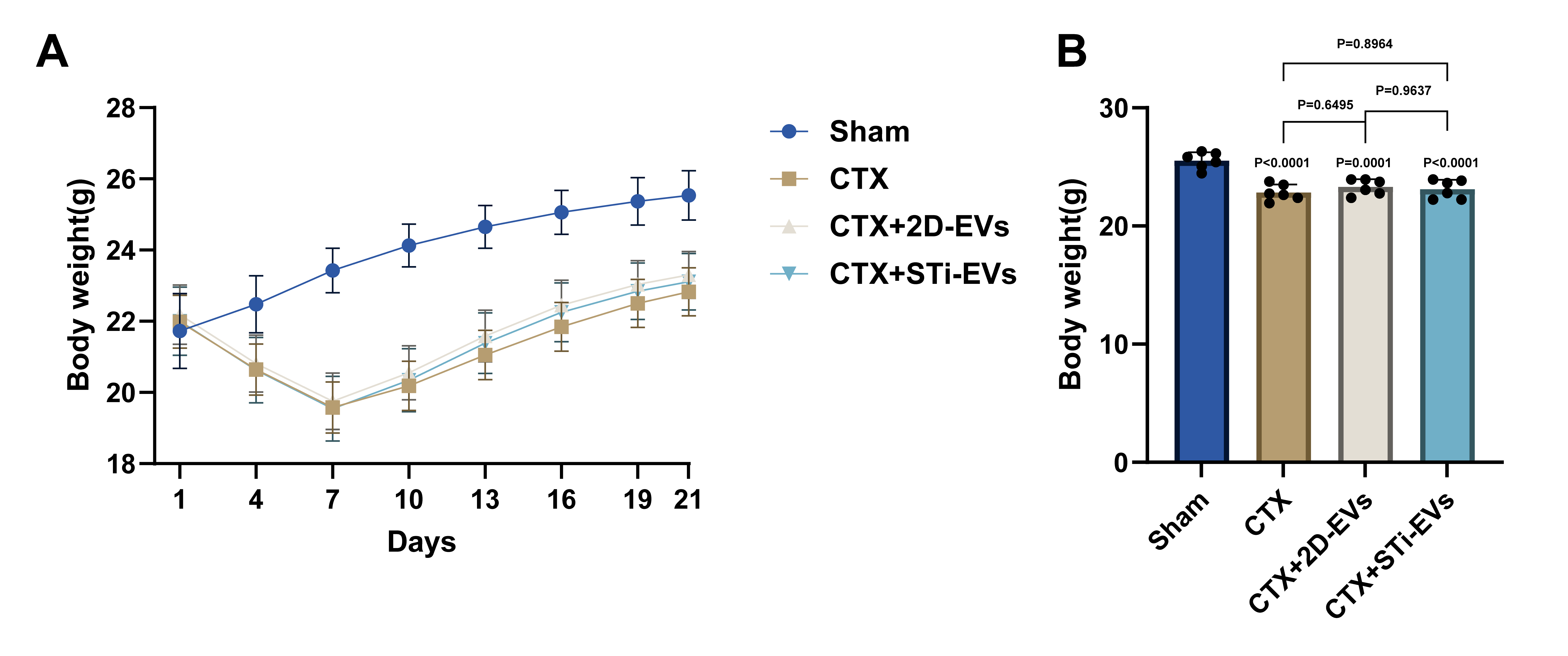


**Figure S3. Body weight monitoring in the CTX mouse model.** (A) Longitudinal body weight changes from day 1 to day 21 in sham and CTX-treated mice with or without EV treatment. (B) Comparison of body weight at day 21 among groups.

**Figure S4. Representative H&E staining of major organs from mice after treatment.** Representative hematoxylin and eosin (H&E) staining images of the lung, heart, liver, spleen, and kidney harvested from mice in the Sham, CTX, CTX + 2D-EVs, and CTX + STi-EVs groups at day 21. Scale bar = 50 μm. n = 6 per group.


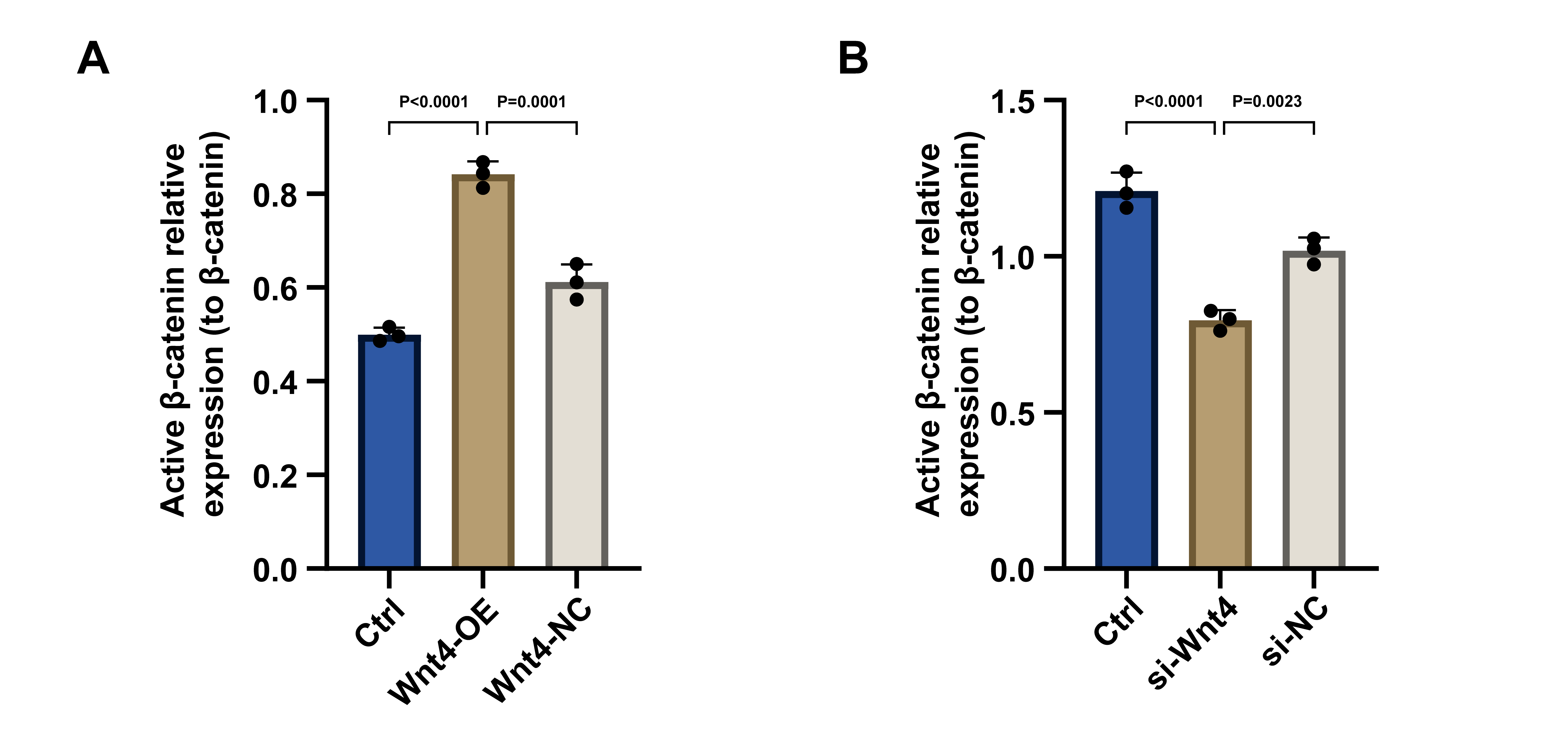
**Figure S5. Same-lane ratio analysis of active β-catenin relative to total β-catenin.** (A) Re-analysis of the Western blot data from the Wnt4 overexpression experiment shown in Fig. 7C–D, expressed as the active β-catenin/total β-catenin ratio within the same lane. (B) Re-analysis of the Western blot data from the Wnt4 silencing experiment shown in Fig. 8C–D, expressed as the active β-catenin/total β-catenin ratio within the same lane.


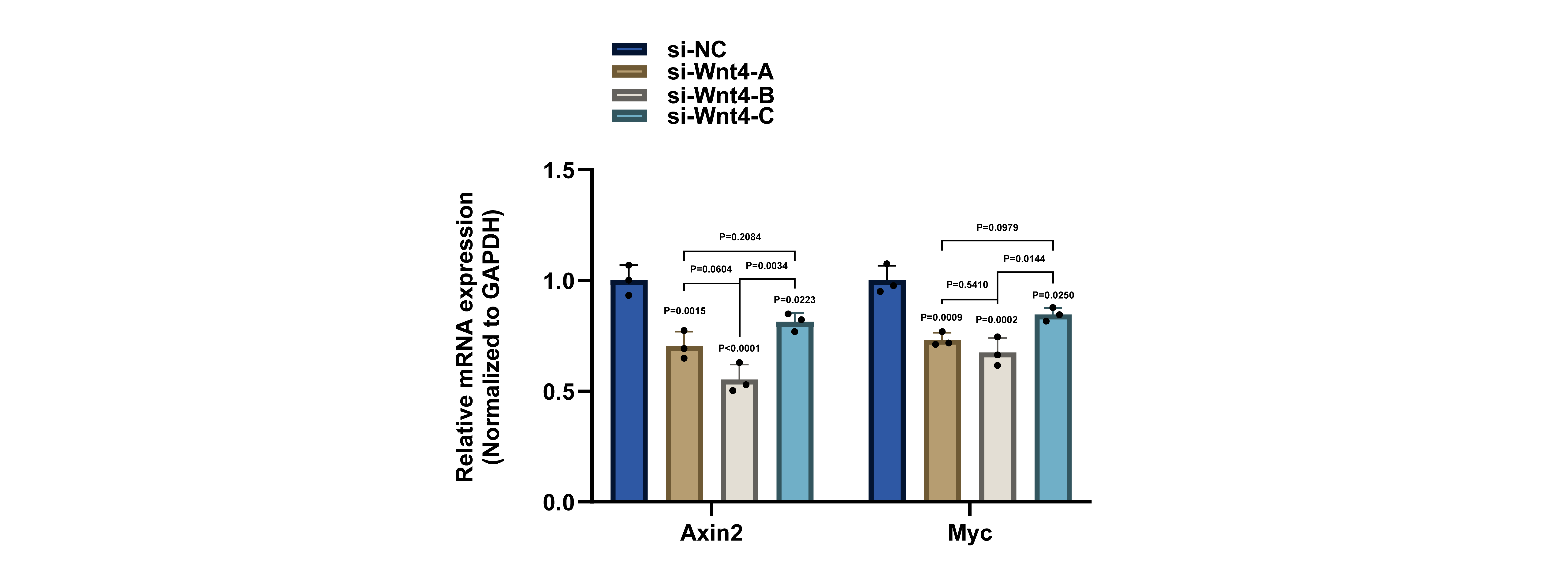


**Figure S6. Validation of si-Wnt4 sequences by downstream canonical Wnt/β-catenin readouts in TM3 cells.** qPCR analysis of the canonical Wnt/β-catenin target genes Axin2 and Myc after transfection of TM3 cells with three independent Wnt4 siRNAs (si-Wnt4-a, si-Wnt4-b, and si-Wnt4-c).


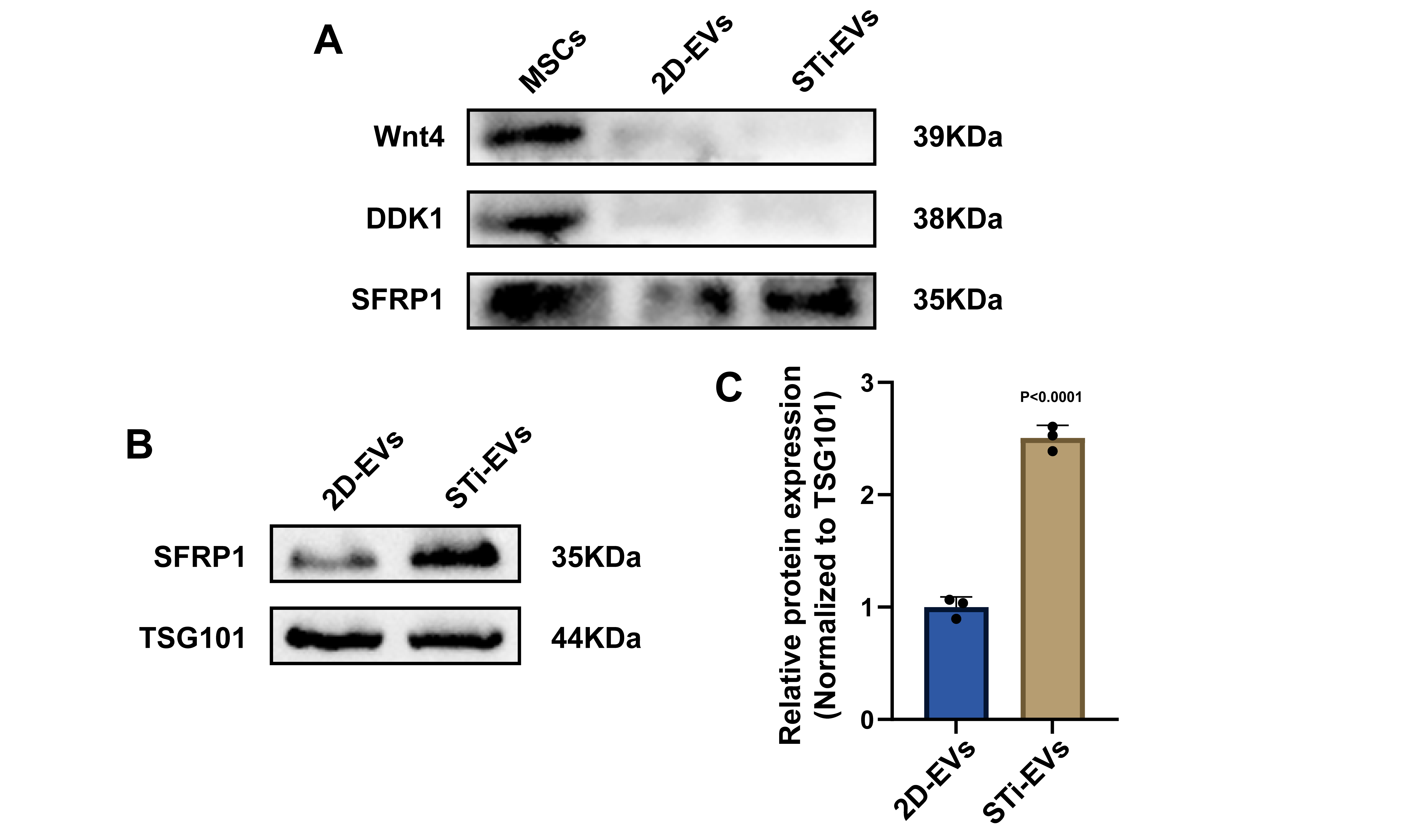
**Figure S7. EV-marker-normalized analysis of SFRP1 enrichment in STi-EVs.** (A)Western blot analysis of Wnt4, DKK1, and SFRP1 in hUMSCs, 2D-EVs, and STi-EVs under equal protein loading. hUMSCs were included as the cell-source control. (B) Western blot analysis of SFRP1 in 2D-EVs and STi-EVs with TSG101 included as an EV-associated normalization marker. (C) Densitometric quantification of SFRP1 protein expression normalized to TSG101. n = 3 per group.


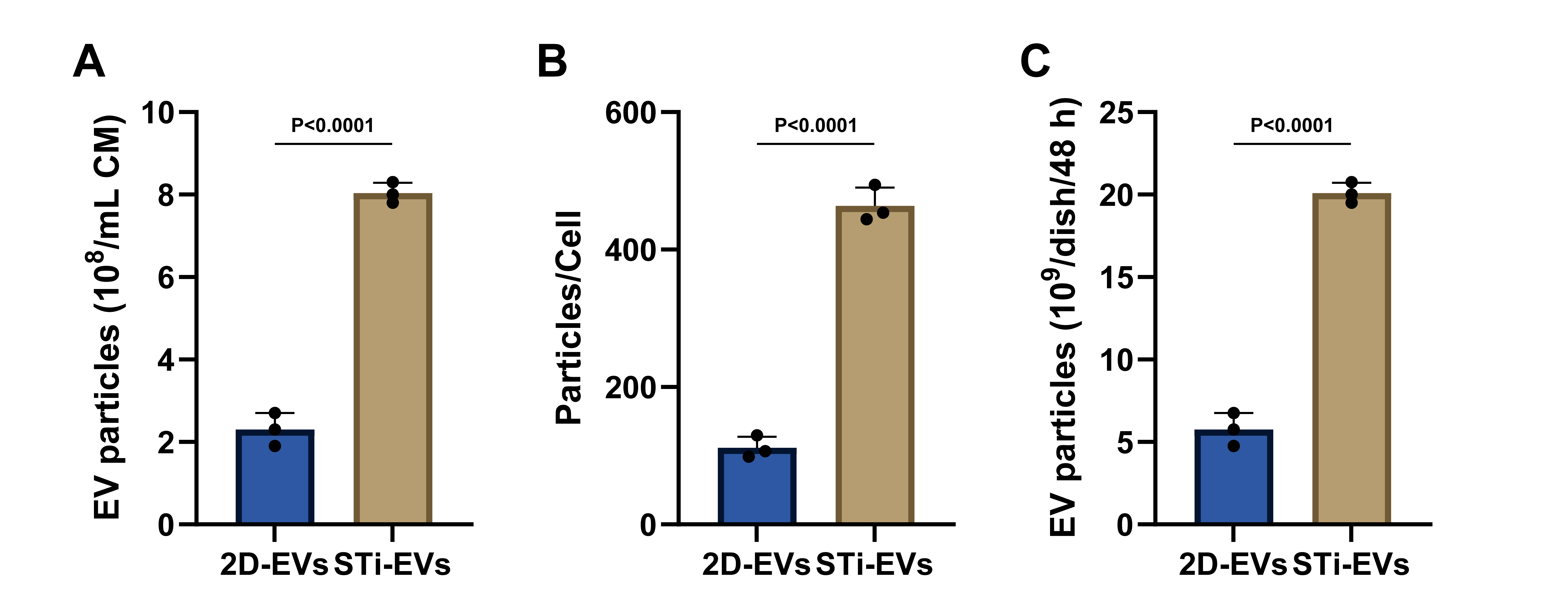


**Figure S8. Quantitative productivity analysis of 2D-EVs and STi-EVs generated in the present study.** (A) EV particle yield per mL conditioned medium (CM). (B) EV particle yield per cell. (C) EV particle yield per dish per 48 h. n = 3 per group.


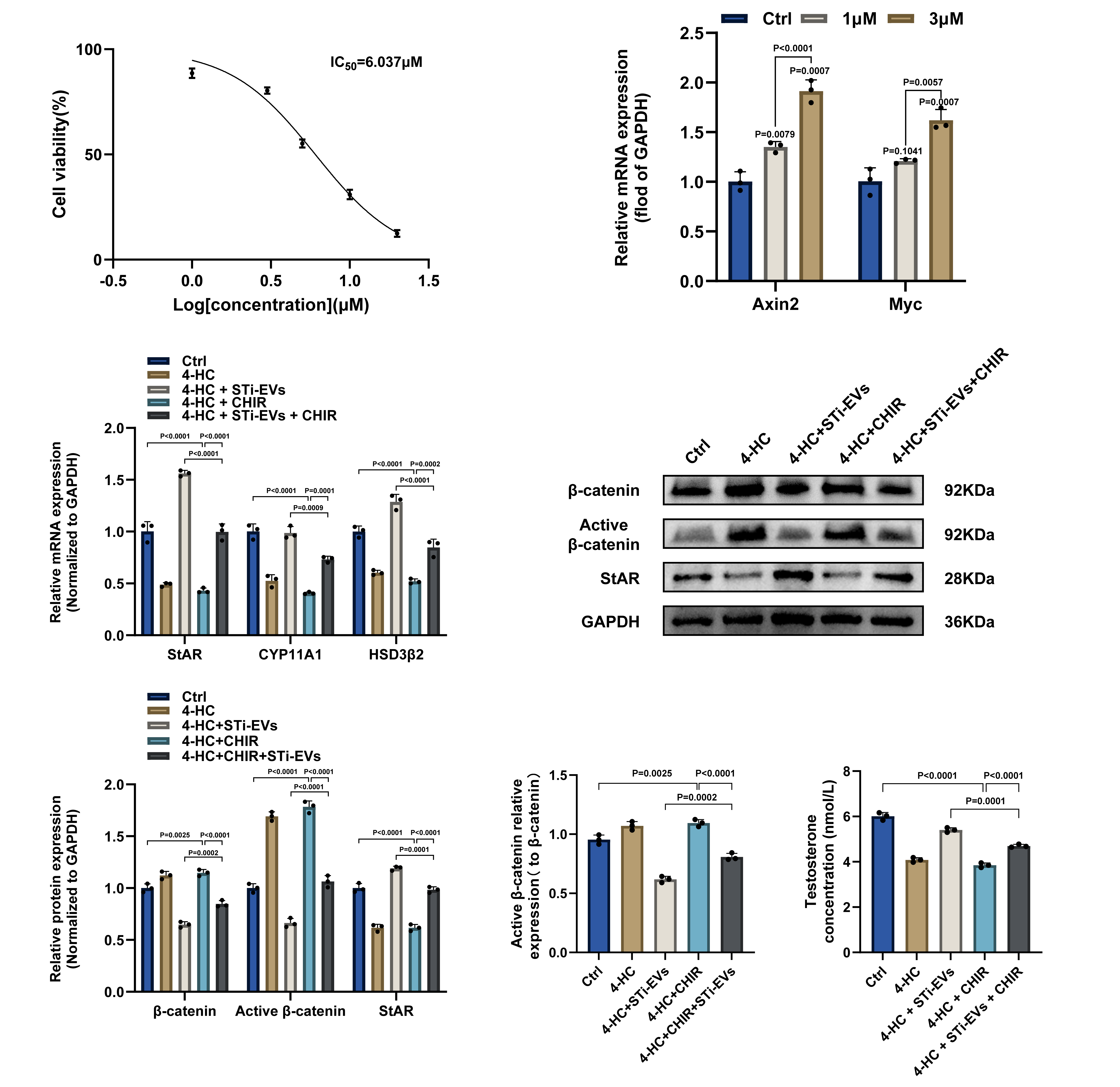


**Figure S9. Pharmacological stabilization of β-catenin by CHIR-99021 attenuates STi-EV-mediated restoration of steroidogenesis in 4-HC-injured TM3 cells.** (A) CCK-8 dose–response analysis of TM3 cell viability after treatment with increasing concentrations of CHIR-99021. (B) qPCR analysis of canonical β-catenin target genes Axin2 and Myc after treatment with 1 μM or 3 μM CHIR-99021. (C) qPCR analysis of steroidogenesis-related genes StAR, CYP11A1, and HSD3β2 in TM3 cells subjected to the indicated treatments. (D) Representative Western blot images showing β-catenin, active β-catenin, and StAR expression after the indicated treatments. (E) Densitometric quantification of β-catenin, active β-catenin, and StAR protein expression. (F) Same-lane ratio analysis of active β-catenin relative to total β-catenin. (G) Testosterone concentration in the culture supernatant measured by ELISA after the indicated treatments. n = 3 per group.
